# Discovery and Pharmacological Evaluation of STEAP4 as a Novel Target for HER2 Overexpressing Breast Cancer

**DOI:** 10.3389/fonc.2021.608201

**Published:** 2021-03-26

**Authors:** Ioanna-Maria Orfanou, Orestis Argyros, Andreas Papapetropoulos, Sofia Tseleni-Balafouta, Konstantinos Vougas, Constantin Tamvakopoulos

**Affiliations:** ^1^ Center for Clinical, Experimental Surgery and Translational Research, Biomedical Research Foundation of the Academy of Athens, Athens, Greece; ^2^ Laboratory of Pharmacology, Faculty of Pharmacy, National and Kapodistrian University of Athens, Athens, Greece; ^3^ Department of Pathology, School of Medicine, National and Kapodistrian University of Athens, Athens, Greece; ^4^ Proteomics Laboratory, Division of Biotechnology, Biomedical Research Foundation of the Academy of Athens, Athens, Greece

**Keywords:** STEAP4, novel pharmacological targets, HER2+ breast cancer, GeLC-MS/MS proteomics, membrane proteins, co-administration schemes

## Abstract

Breast cancer (BC) is a highly heterogeneous disease encompassing multiple subtypes with different molecular and histopathological features, disease prognosis, and therapeutic responses. Among these, the Triple Negative BC form (TNBC) is an aggressive subtype with poor prognosis and therapeutic outcome. With respect to HER2 overexpressing BC, although advanced targeted therapies have improved the survival of patients, disease relapse and metastasis remains a challenge for therapeutic efficacy. In this study the aim was to identify key membrane-associated proteins which are overexpressed in these aggressive BC subtypes and can serve as potential biomarkers or drug targets. We leveraged on the development of a membrane enrichment protocol in combination with the global profiling GeLC-MS/MS technique, and compared the proteomic profiles of a HER2 overexpressing (HCC-1954) and a TNBC (MDA-MB-231) cell line with that of a benign control breast cell line (MCF-10A). An average of 2300 proteins were identified from each cell line, of which approximately 600 were membrane-associated proteins. Our global proteomic methodology in tandem with invigoration by Western blot and Immunofluorescence analysis, readily detected several previously-established BC receptors like HER2 and EPHA2, but importantly STEAP4 and CD97 emerged as novel potential candidate markers. This is the first time that the mitochondrial iron reductase STEAP4 protein up-regulation is linked to BC (HER2+ subtype), while for CD97, its role in BC has been previously described, but never before by a global proteomic technology in TNBC. STEAP4 was selected for further detailed evaluation by the employment of Immunohistochemical analysis of BC xenografts and clinical tissue microarray studies. Results showed that STEAP4 expression was evident only in malignant breast tissues whereas all the benign breast cases had no detectable levels. A functional role of STEAP4 intervention was established in HER2 overexpressing BC by pharmacological studies, where blockage of the STEAP4 pathway with an iron chelator (Deferiprone) in combination with the HER2 inhibitor Lapatinib led to a significant reduction in cell growth *in vitro*. Furthermore, siRNA mediated knockdown of STEAP4 also suppressed cell proliferation and enhanced the inhibition of Lapatinib in HER2 overexpressing BC, confirming its potential oncogenic role in BC. In conclusion, STEAP4 may represent a novel BC related biomarker and a potential pharmacological target for the treatment of HER2 overexpressing BC.

## Introduction

Breast cancer (BC) is one of the most frequently diagnosed malignancies and the leading cause of cancer-related death in women worldwide, with more than one million estimated new cases and nearly five thousand related deaths each year (1, 2). It is also the second leading cause of cancer-related mortalities globally trailing lung cancer (2). BC is a very heterogeneous disease and based on gene expression profiling as well as immunohistochemistry it can be classified into five major molecular subtypes, Luminal A and Luminal B subtypes that express estrogen and progesterone receptors (ER and PR), HER2 overexpressing (HER2+) subtype which is characterized by the overexpression of the HER2 receptor, Triple Negative (TN) subtype that lacks expression of ER, PR and HER2 and normal-like that is ER and PR positive and HER2 negative (3). While Luminal and normal-like subtypes carry a good prognosis and therapeutic response, HER2+ (about 20% of all BC) and TN subtypes (15-20% of all BC) are associated with poor prognosis, frequent disease relapse and poor therapeutic outcome (4, 5). Although established targeted therapies like trastuzumab and Lapatinib have considerable efficacy in HER2+ BC patients, inherent and acquired drug resistance results in relapse and progression of the disease (6, 7). With respect to TNBC, the first targeted agents, including poly (ADP-ribose) polymerase inhibitors (olaparib and talazoparib) and an immune checkpoint inhibitor (atezolizumab), have been very recently approved for the treatment of TNBC patients (8, 9). However, up to now the mainstay of treatment has been chemotherapy alone (10, 11). Consequently, new targeted diagnostic or therapeutic agents for HER2+ BC and TNBC are urgently needed to improve disease outcomes, which was the main focus of the current study.

We decided to focus our analysis in membrane protein targets due to their central role in all physiological functions, such as cell signalling, cell-cell interactions and cell homeostasis and over all implication in the development and progression of a variety of human cancers (12, 13). However, due to their relatively low abundance and hydrophobicity, membrane protein analysis is a common inherent proteomic challenge. To overcome potential analytical difficulties, we employed a commercially available membrane protein extraction kit to enrich integral membrane and membrane-associated proteins from different BC cell lines and the enriched membrane proteins were subjected to high resolution Mass Spectrometry (MS) analysis.

MS-based proteomics is currently the backbone of cancer biomarker discovery as the technology affords the high throughput study of proteins from complex biological samples aiming to the investigation of their ontology, classification, expression levels and properties (14, 15). The development of protein-level separation by 1D-SDS-PAGE followed by high resolution LC-MS/MS analysis referred to as GeLC-MS/MS, provides a powerful analytical tool for recovering and studying low-abundance, hydrophobic and basic membrane proteins (14, 16, 17). The GeLC-MS/MS approach has been applied to several types of cancer, including BC (18–22).

In this study, we leveraged on a label-free GeLC-MS/MS technique to compare the membrane proteomes of two representative and regularly used epithelial BC cell lines, such as the HCC-1954 (HER2+) and the MDA-MB-231 (TNBC) to a breast benign control cell line, MCF-10A. Several hits of potential interest were generated with CD97 and the iron reductase six-transmembrane epithelial antigen of prostate 4 (STEAP4) distinguished as the most novel and promising of these hits. STEAP4 protein was selected for further downstream analysis by employing Immunohistochemical (IHC) analysis on tumors derived from xenografted mice inoculated with the BC cell lines and on two independent BC tissue microarrays (TMAs). We also performed proof-of-principle functional studies on STEAP4 to assess its involvement in cancer pathophysiology. The STEAP4 pathway was pharmacologically targeted in BC cells, with an iron chelating drug (Deferiprone) with simultaneous blockage of the HER2 pathway with the tyrosine kinase inhibitor Lapatinib, which demonstrated an additive therapeutic potential involvement of STEAP4 in cancer.

Overall, our data suggest that STEAP4 may constitute a novel BC biomarker or a promising new target for HER2+ BC therapy.

## Materials and Methods

### Cell Culture

The BC cell lines HCC-1954 (CRL-2338), SKBR3 (HTB-30), BT474 (HTB-20), and MDA-MB-231 (HTB-26), and the breast benign epithelial cell line MCF-10A (CRL-10317) were obtained from the American Type Culture Collection (ATCC; Manassas, VA, USA). The HCC-1954, the SKBR3 and the BT474 cells were cultured in RPMI 1640 and the MDA-MB-231 in Dulbecco’s modified Eagle’s (DMEM), both medium were supplemented with 10% fetal bovine serum (FBS) and 1% antibiotics (penicillin/streptomycin). The MCF-10A was cultured in DMEM supplemented with 10% FBS, epidermal growth factor (20 ng/mL), cholera toxin (100 ng/mL and hydrocortisone (0.5 μg/mL). All cells were incubated in a humidified atmosphere at 37°C, 5% CO_2_ and regularly screened for mycoplasma using the MycoAlert™ Mycoplasma Detection Kit (Lonza, USA). All experiments were performed on cell cultures passaged no more than six times from frozen stock vials of passage 20 for HCC-1954 and SKBR3, 15 for BT474, 25 for MDA-MB-231, and 2 for MCF-10A.

### Subcellular Fractionation and Membrane Enrichment Procedure

Enriched membrane fractions from the BC cell lines HCC-1954 and MDA-MB-231 and the breast benign control cell line MCF-10A were prepared and separated from cytosolic fractions using a commercially available kit (Mem-PER Plus Membrane Protein Extraction Kit) according to the manufacturer’s instructions. Briefly, 5 × 10^6^ cells were harvested and the cell suspension was centrifuged at 300 × g for 5 min. Then the cell pellet was washed with 3 mL Cell Wash Solution, centrifuged at 300 × g for 5 min and the supernatant was discarded. The cells were resuspended in 1.5 mL Cell Wash Solution, transferred to small tube and centrifuged again at 300 × g for 5 min. The cell pellet was then mixed with 0.75 mL of Permeabilization Buffer, vortexed briefly to obtain a homogeneous cell suspension and incubated 10 min at 4°C with constant mixing. The permeabilized cells were centrifuged for 15 min at 16,000 × g and the supernatant containing the cytosolic proteins was carefully collected to a new tube and stored at -80°C for future use. Subsequently, the protein pellet was resuspended in 0.5 mL of Solubilization Buffer, incubated at 4°C for 30 min with constant mixing and centrifuged at 16,000 × g for 15 min at 4°C. Finally, the supernatant containing solubilized membrane and membrane-associated proteins was transferred to a new tube and stored at -80°C for further analysis. Meanwhile, whole cell lysate fractions were isolated using the Ripa Buffer cell lysis protocol, as described before. The protein concentrations of the resulting membrane cell lysate fractions were measured using the Bradford assay.

### GeLC-MS/MS Analysis

One-dimensional SDS-PAGE and in-gel trypsin digestion were performed as previously reported (23). Briefly, 10 μg of the enriched membrane fractions from individual cell lines were run in 12% SDS PAGE, and stained with Coomassie Colloidal Blue overnight. Bands were excised from the gels and cut into small pieces (1-2 mm). Gel pieces were destained in 40% acetonitrile and in 50 mM NH_4_HCO_3_, reduced in 10 mM DTE and 100 mM NH_4_HCO_3_, and alkylated in 50 mM IAA and 100 mM NH_4_HCO_3_. Samples were dried using the Savant Speedvac™ concentrator (ThermoFisher Scientific, Waltham, MA, USA) and trypsinized overnight with 600 ng trypsin, using a trypsin stock solution of 10 ng/μL in 10 mM NH_4_HCO_3_. The extraction of peptides was performed with sequential washes of the trypsinized gel pieces with 50 mM NH_4_HCO_3_, followed by two washes with 50% acetonitrile, 5% formic acid for 15 min at room temperature, with agitation. The extracted peptides were dried using the Savant Speedvac™ concentrator and analyzed by nano-LC-MS/MS analysis.

### LC-MS/MS Analysis

All LC-MS/MS experiments were performed on a Dionex Ultimate 3000 UHPLC system coupled with the high resolution nano-ESI Orbitrap-Elite mass spectrometer (Thermo Finnigan, Bremen, Germany), as previously described (24). Briefly, 5 μL corresponding to 5 μg of the peptide mixture were analyzed on a nanoflow system (Dionex™, Camberly, UK). After loading on a Dionex 0.1×20 mm, 5 μm C18 nanotrap column at a flow rate of 5 μL/min in 98% mobile phase A (0.1% formic acid) and 2% mobile phase B (100% acetonitrile, 0.1% formic acid), the sample was eluted into an Acclaim PepMap C18 nanocolumn 75 μm × 50 cm (Dionex™, Sunnyvale, CA, USA), 2 μm 100 Å, at a flow rate of 0.3 μL/min. The trap and the nanoflow column were maintained at 35°C. The samples were eluted with a gradient of solvent A: solvent B starting at 2%B for 10 min, rising to 5%B at 11 min, 15%B at 73 min and 55%B at 95 min. The column was then washed and re-equilibrated prior to injection of the next sample. The eluant was ionized using a Proxeon nanospray ESI source, operating in positive ion mode into an Orbitrap Elite FTMS (Thermo Finnigan, Bremen, Germany). Ionization voltage was at 2.2 kV and the capillary temperature was at 250°C. The mass-spectrometer was operated in MS/MS mode scanning from 300 to 2200 amu. The resolution of ions in MS1 was 60,000 and 15,000 for HCD MS2. The top 10 multiply charged ions were selected from each scan for MS/MS analysis using HCD at 33% collision energy. Data analysis was performed with Proteome Discoverer 1.4 software package (Thermo Finnigan), using the Sequest search engine and the Uniprot human reviewed database, updated on May 30, 2016, including 20,204 entries. The search was conducted using carbamidomethylation of cysteine as static and oxidation of methionine as dynamic modifications. Two missed cleavage sites, a precursor mass tolerance of 10 ppm and fragment mass tolerance of 0.05 Da were allowed. False discovery rate (FDR) validation was based on q value: target FDR (strict): 0.01, target FDR (relaxed): 0.05. SEQUEST results were filtered for false-positive identifications. The MS proteomics data have been deposited to the ProteomeXchange Consortium *via* the PRIDE partner repository with the data set identifier PXD021819.

### LC-MS/MS Quantification Analysis

Quantification analysis was performed using a label-free approach based on the peak area intensities of identified proteins (24, 25).

The peak area intensity measuring and comparing is the most widely used label-free quantification method which is based on the precursor signal intensity as determined by the extracted ion chromatogram (XIC). Precursor signal intensity is strongly correlated with peptide abundance in a specific sample (26). The peak area-based quantification uses precursor ions to assess the relative abundance of identified proteins in the label-free data. Precursor ion chromatogram for each peptide is extracted from individual LC-MS/MS runs and their peak area is calculated during data processing in Proteome Discoverer (Thermo Scientific) by using the Precursor Ions Area Detector node. For the accuracy of protein identification, the individual aligned peak must correspond to its precursor ion, retention time, charge state, and fragmented ion. After the alignment process, identified peptides from each sample are measured and normalized for their calculated peak areas and then equated for a comparative amount of protein. Protein abundance in each sample was calculated as the sum of all normalized protein areas.

Area intensity normalization for each identified protein based on total ion count is a necessary step to eliminate bias in signal intensity (27). The area intensity for each protein was normalized according to the following formula: Normalized Area = (Protein Area/Total Area) × 10^6^. Finally, statistical analysis and comparison of Normalized peak area intensities was performed in order to determine the significance of changes between the biological samples (e.g., cell line A versus cell line B). Comparison of the area intensity for each identified protein from multiple LC-MS/MS datasets is suitable for clinical biomarker discovery, which normally requires high-throughput sample analysis (28, 29).

### Western Blot Analysis

Cells were lysed in ice-cold RIPA lysis buffer (50 mM Tris-HCl at pH 7.5, 150 mM NaCl, 1 mM EGTA, 5 mM Na_2_EDTA, 0.1% SDS, 1% NP-40, 0.5% sodium deoxycholate, 8 mM sodium fluoride, 1 mM sodium orthovanadate) containing protease and phosphatase inhibitor cocktail (Roche, UK). Protein concentration was determined using the Pierce BCA assay kit (Pierce, Rockford, IL, USA) according to the manufacturer’s specifications. Equal amounts of protein extracted from the HCC-1954, the MDA-MB-231, the SKBR3, the BT474, and the MCF-10A cell lysates (10 μg of protein/cell line), along with NuPAGE Sample Reducing agent and NuPAGE LDS Sample Buffer were then separated by 10% SDS-PAGE gel electrophoresis and transferred onto polyvinylidene difluoride (PVDF) membranes. After blocking with 5% non-fat dried milk in TBS-T buffer (20 mM Tris, pH 7.6, 137mM NaCl, 0.1% Tween 20) for 1 h at room temperature, the membranes were incubated with primary antibodies overnight at 4 ^0^C and then with horseradish peroxidase (HRP)-conjugated secondary antibodies for 2h at room temperature. The primary antibodies used were: mouse monoclonal anti-ErbB2 (1:1000 dilution; cat. no. ab8054, Abcam, UK), rabbit monoclonal anti-EPHA2 (1:3000 dilution; cat. no. 6997S, Cell Signaling Technology, USA), rabbit monoclonal anti-CD97 (1:1000 dilution; cat. no. ab108368, Abcam, UK) and rabbit polyclonal anti-STEAP4 (1:2500 dilution; cat. no. 11944-1-AP, Proteintech, USA). For normalization of protein concentration, the rabbit monoclonal anti-β-actin (1:10000 dilution; cat. no. ab190476, Abcam, UK) was used as loading control. The secondary antibodies used were: horseradish peroxidase (HRP)-conjugated goat anti-rabbit or horse anti-mouse IgG (1:2000 dilution; cat. no. 7074S and 7076S, Cell Signaling Technology, USA). Reactive protein bands were visualized by incubation of the membranes with Enhanced Chemiluminescence substrate (GE Healthcare, UK) and exposure to x-ray films. All Western blot analyses were repeated at least three times.

### Immunofluorescence (IF) Analysis

HCC-1954, MDA-MB-231, SKBR3, BT474 and MCF-10A cells were grown onto coverslips in 24-well plate at a density of 1 × 10^4^ cells at 37°C. The next day, the culture media was discarded, cells were rinsed with PBS three times and then fixed with 100% methanol at -20°C for 10 min. The cells were further washed twice with ice-cold PBS (5 min per wash) to remove fixative agent. Subsequently, 200 μL of blocking buffer (5% goat serum, 0.1% Triton in PBS) was added to the cells and incubated for 1 h at room temperature to block nonspecific binding with antibody. After blocking, the cells were incubated with the following primary antibodies overnight at 4°C: rabbit polyclonal anti-STEAP4 antibody (1:500 dilution; cat. no. 11944-1-AP, Proteintech, USA), mouse monoclonal anti-ErbB2 (1:100 dilution; cat. no. ab8054, Abcam, UK), rabbit monoclonal anti-EphA2 (1:200 dilution; cat. no. 6997S, Cell Signaling Technology, USA) and rabbit monoclonal anti-CD97 (1:200 dilution; cat. no. ab108368, Abcam, UK). Cells were then washed twice with ice-cold PBS, and incubated with the secondary antibody conjugated to Alexa Fluor 568 donkey anti-rabbit IgG (1:500 dilution; cat. no. ab175470, Abcam, UK) for 2 h in the dark at room temperature. The coverslips containing the cells were then washed twice with ice-cold PBS and mounted on glass slides with ProLong Gold anti-fade reagent containing DAPI (1.5 μg/mL) (Invitrogen Inc., Eugene, OR, USA). Dapi was used to stain the cell nucleus. Immunofluorescent stained cells were dried overnight in the dark at 4°C and the analyses were made using the fluorescence microscope (Leica Microsystems) with a 400 × objective. To allow direct comparisons, all images were captured using the same parameters. The fluorescence intensity was quantified using ImageJ software.

### Immunohistochemistry (IHC) Analysis of Xenografts and Tissue Microarrays

Formalin fixed, paraffin-embedded tissues derived from xenografted mice inoculated with the HCC-1954 and the MDA-MB-231 cell lines were obtained for IHC analysis of STEAP4 expression as previously described (30). For the generation of the xenograft tumor sections, female NOD/SCID mice were injected in each flank with 3 × 10^6^ HCC1954 or MDA-MB-231 cells. When tumors reached 100–150 mm^3^, 2-hydroxypropyl β-Cyclodextrin (HP-b-CD) was administered daily by intraperitoneally (IP) injection. After 18 days, tumors were excised, fixed in neutral buffered formalin, paraffin embedded and sectioned. All animal procedures for the data in xenografted mice were approved by the Bioethical Committee of BRFAA based on the European Directive 86/609.

Two human BC tissue microarray slides (catalog # BRC961; Pantomics, Rockville, MD and catalog # BR251c; US Biomax, Rockville, MD) containing duplicate cores from a total of 36 different cases of BC with 12 cases of normal and benign tumor tissues and 6 quadrupole cases of breast invasive ductal carcinoma with matched adjacent normal breast, respectively, were used for expression studies of STEAP4 in clinical samples. For the TMAs, all human tissues were collected under HIPPA approved protocols, as described in the the providers of the tissue microarrays, US Biomax, Inc. (https://www.biomax.us/FAQs), and US Pantomics, Inc. (https://www.pantomics.com/technical-faqs). The characteristics of the samples derived from TMAs can be viewed in the Pantomics and Biomax Web site (https://www.pantomics.com/Masterdatas/ArrayCatalog/Tissue-Arrays/BRC961 and https://www.biomax.us/tissue-arrays/Breast/BR251c). IHC of xenografts and TMAs was performed according to the Biomax standard protocols (https://www.biomax.us/Support). In brief, paraffin-embedded xenograft and TMA sections were warmed in a 60°C oven for about 10-30 min, were deparaffinized in xylene, rehydrated in a graded series of ethanol solutions (100, 95, 70 and 50%), and subjected to antigen retrieval treatment in Tris-EDTA buffer (pH 9.0). After quenching of endogenous peroxidase activity with 3% H_2_O_2_ for 15 min and blocking with normal serum for 1 h, sections were incubated with primary rabbit polyclonal anti-STEAP4 antibody (1:150 dilution; cat. no. 11944-1-AP, Proteintech, USA) overnight at 4°C. Slides were then incubated with biotinylated secondary anti-rabbit IgG (H+L) antibody (1:200 dilution; cat. no. 14708S, Cell Signaling, UK) for 30 min at 37°C, followed by Streptavidin-HRP (1:200 dilution; cat. no. 3999S, Cell Signaling, UK). Sections were finally developed with 3, 3’-diaminobenzidine (DAB) substrate solution (DAKO, CA) as chromogen and counterstained with hematoxylin for microscopic visualization. A scale of 1 to 4, representing negative (1), weak (2), moderate (3) and strong (4) staining, respectively, was used to grade the intensity of staining. The stained sections were evaluated by a certified pathologist, who was blinded to the information of sections during microscopic examination and evaluation. Specimens assigned scores of 1 to 2 were considered to have low expression, whereas those with scores 3 to 4 were regarded as having high expression.

### Cell Viability Assays

For the pharmacological studies, Lapatinib (cat. no. HY-50898) was purchased from MedChem Express and Deferiprone (3-hydroxy-1,2-dimethyl-4(1H)-pyridone) from Sigma-Aldrich.

HCC-1954, SKBR3, BT474 and MCF-10A cells were seeded at a density of 5 × 10^3^ cells per well on 96-well plates. After 24 h incubation (37°C, 5% CO_2_), the cell medium was removed, and the cells were treated with various concentrations of Lapatinib (10 nM-30 μM) and deferiprone (10 nM-30 μM), either alone or in combination for 72 h. The medium was then removed and the 3-(4,5-dimethylthiazol-2-yl)-2,5-diphenyltetrazolium bromide (MTT) solution (0.3 mg/mL in PBS) was added to cells for 3 h, after which the MTT solution was removed and the formazan crystals were dissolved in 100 μL DMSO. The optical density was measured at 570 nm and at a reference wavelength of 650 nm using an absorbance microplate reader (SpectraMax 190, Molecular Devices, Sunnyvale, CA, USA). The 50% cytostatic concentration (IC_50_) was calculated based on a four-parameter logistic equation using GraphPad Prism 5 (GraphPad Software, Inc.).

### siRNA Transfection and Cell Viability Assays—Knockdown of STEAP4 in HCC-1954 Cells

Three different siRNA duplexes specific for STEAP4: A-siRNA (cat. no. SR312522A), B-siRNA (cat. no. SR312522B), C-siRNA (cat. no. SR312522C) and a Universal Scrambled negative control siRNA (NC-siRNA) duplex (cat. no. SR30004) were purchased from OriGene (OriGene, Rockville, MD). HCC-1954 cells were transfected with a final concentration of 10 nM siRNA, using siTRAN 2.0 transfection reagent (OriGene, Rockville, MD), according to the manufacturer’s protocol. Control cells were mock transfected with transfection reagents. HCC-1954 cells (2 x 10^5^ per well) were seeded in 6-well plates and cultured until they reached 60% - 70% confluency. The cells were incubated with transfection mixtures containing 10 nM of STEAP4-siRNA or NC-siRNA (non-silencing of STEAP4) for 14 h and then the culture supernatant was replaced with fresh culture medium. The cells were harvested after 48 h and evaluated for their silencing efficiency using Western Blot, as described above.

To determine the proliferative ability of cells, HCC-1954 cells at a density of 5 × 10^3^ cells per well were cultured in 96-well plates. The cells were initially transfected with STEAP4-siRNA or NC-siRNA. After 24, 48 and 72 h of transfection, the cell medium was removed and MTT solution (0.3 mg/mL in PBS) was added to cells for 3 h at 37°C. The optical density was measured at 570 nm and at a reference wavelength of 650 nm using an absorbance microplate reader (SpectraMax 190, Molecular Devices, Sunnyvale, CA, USA).

To investigate the effect of siRNA on Lapatinib IC_50_ values, HCC-1954 cells were seeded into 96-well plates (5 × 10^3^ cells/well) and allowed to attach for 24 h (37°C, 5% CO2). Then, cells were treated with 10 nM of STEAP4-siRNA or NC-siRNA for 14 h at 37°C in a 5% CO_2_ incubator. After incubation, the culture supernatant was replaced with fresh culture medium containing serum and different concentrations of Lapatinib (10 nM-30 μM) for a total time of 72 h. After incubation, the cell viability was detected using the same MTT assay described above.

### Statistical Analysis

The results presented herein are expressed as mean ± SD. Correlations between STEAP4 expression and clinicopathological parameters were determined using the Chi-square test. Statistical analyses and calculation of all IC_50_’s were performed by GraphPad software. Each point was the result of three independent experiments performed in triplicate for statistical analysis. Statistical significance was determined using the Student’s t-test. A P value of less than 0.05 was considered significant.

## Results

### Membrane Protein Enrichment and Isolation

**Figure 1:**
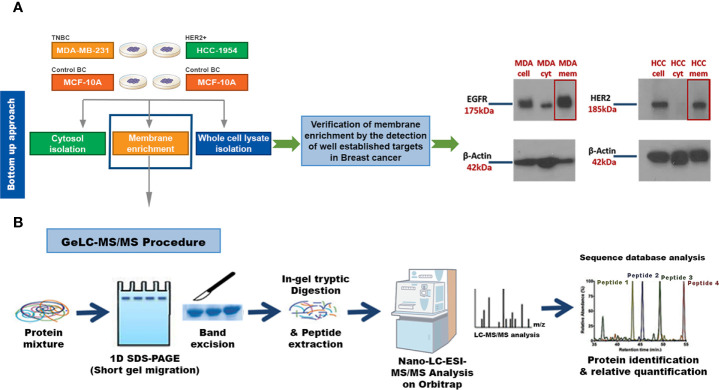
Experimental workflow for identification of candidate Breast Cancer (BC) biomarkers or drug targets using a strategy that combines a membrane enrichment protocol with GeLC-MS/MS technique. **(A)** Membrane enriched, cytosolic and whole cell lysate fractions from the breast cancerous MDA-MB-231 (MDA) and HCC-1954 (HCC) cell lines were isolated and analyzed by Western blot, using well established BC membrane targets (HER2 and EGFR), to verify the success of the membrane enrichment. **(B)** Membrane enriched fractions were then subjected to 1D SDS-PAGE gel electrophoresis, in-gel trypsin digestion, and tandem mass spectrometric analysis on a high resolution Orbitrap. Protein identification and quantification was performed using bioinformatic tools (Sequest database search). Cell, whole cell lysate fraction; cyt, cytosolic fraction; mem, membrane fraction.

Our key methodological principle was to increase the threshold of sensitivity to ensure that even low-abundant cell membrane proteins in aggressive subtypes of BC could be detected. Thus, our initial step was to perform an efficient subcellular fractionation of three well characterized BC epithelial cell lines: HCC-1954 (HER2 overexpressing), MDA-MB-231 (TNBC) and MCF-10A (benign control). Enriched membrane fractions were isolated and separated from the cytosolic fractions using a commercially available kit, whereas whole cell lysate fractions were prepared using the Ripa Buffer cell lysis protocol (**Figure 1A**). The whole uncropped images of the original Western blots for **Figure 1A** are provided in **Supplementary Figure 4**. To verify the successful enrichment of the membrane isolation protocol, we performed Western blot analysis of well-established BC biomarkers, the EGFR and HER2, which served as our positive control. As shown in **Figure 1A** strong expression of EGFR and HER2 was observed in the isolated membrane fractions compared to the cytosolic fractions.

### Proteomic Analysis of the Membrane Enriched Fractions

Following the successful fractionation, a global proteomic profiling was performed in order to identify new pharmacological targets or biomarkers for HER2+ and TNBC. For such a demanding proteomic analysis we utilized the Global Discovery-based GeLC-MS/MS approach on a high resolution Orbitrap Mass analyzer. The technique is based on the initial separation of the protein mixture by one-dimensional SDS-page gel electrophoresis followed by in-gel tryptic digestion and finally on the Mass Spectrometric and Bioinformatic analysis of the peptide mixture (**Figure 1B**). Four biological replicates were used for each cell type, and from the analysis of replicates of the HCC-1954 cell line, an average of 2200 proteins were identified, whereas an average of 2500 proteins were identified from the MDA-MB-231 cell line and an average of 2100 proteins were identified from the MCF-10A cell line. Since we were interested in easily accessible cell membrane and membrane associated proteins that can be exploited for drug design and for diagnostic purposes, the list of identified proteins was refined for the selection of membrane associated proteins based on their subcellular location (Source: Uniprot database http://www.uniprot.org). Given the heterogeneous nature of BC, only proteins that were present consistently in 75% of cancer biological replicates and at above the limit of detection (Normalized Area ≥ 70) were chosen for downstream analysis. Refining the search with those criteria, a total of 536, 641 and 604 membrane-associated proteins were selected from the HCC-1954, the MDA-MB-231 and the MCF-10A cell lines, respectively.

**Figure 2:**
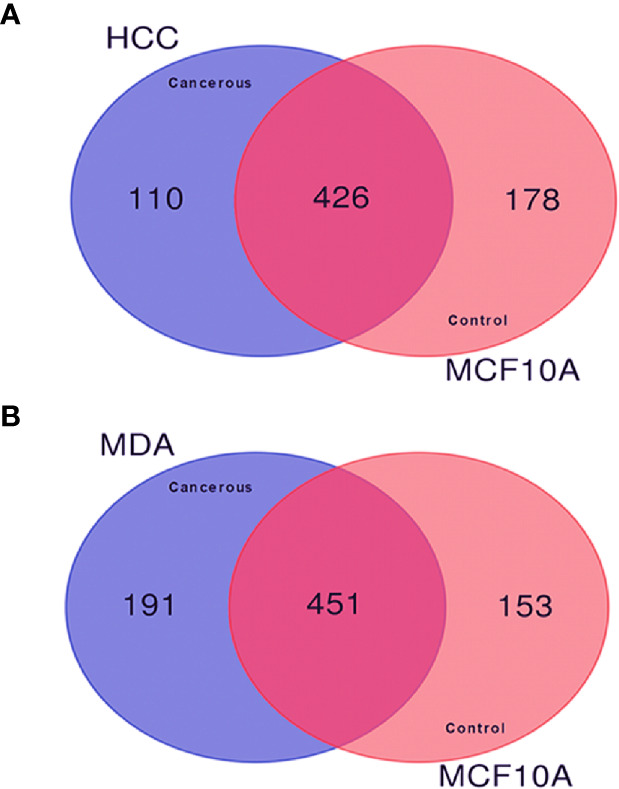
Comparison of the membrane-associated proteins identified in breast cancer (HCC-1954 and MDA-MB-231) versus benign control (MCF-10A) cell lines by GeLC-MS/MS analysis. Proteins from 4 biological replicates of each breast cancer cell line were combined for this comparison. **(A)** Venn diagram depicting the comparison of HCC-1954 vs. MCF-10A cells, of which 426 proteins were commonly identified, while 110 and 178 proteins were uniquely identified in HCC-1954 and MCF-10A, respectively. **(B)** Venn diagram depicting the comparison of MDA-MB-231 vs. MCF-10A cells, of which 451 proteins were commonly identified, while 191 and 153 proteins were uniquely identified in MDA-MB-231 and MCF-10A, respectively.

In order to investigate the quantitative similarities and differences in protein expression resulting from malignant transformation of the breast epithelium, comparative proteomic analysis was performed among the described BC cell lines. The Venn diagrams in **Figures 2A, B** display the comparisons of the membrane-associated proteins identified between each BC cell line (HCC-1954 and MDA-MB-231) and the benign control cell line (MCF-10A). In the comparison of HCC-1954 versus MCF-10A cells, 426 proteins were common, whereas 110 and 178 proteins were unique in the HCC-1954 and the MCF-10A, respectively. Also, in the MDA-MB-231 versus the MCF-10A comparison there were 451 common identifications, while there were 191 and 153 unique protein identifications in the MDA-MB-231 and the MCF-10A, respectively. The original lists of all the membrane-associated proteins found in the HCC-1954 cells compared to the MCF-10A cells and in the MDA-MB-231 cells compared to the MCF-10A cells along with their Normalized Areas are provided in **Supplementary Tables 1** and **2**, respectively.

### Selection of Promising Candidate Drug Targets or Biomarkers for HER2+ or TNBC

Quantitative comparison of HCC-1954 (HER2+) and MDA-MB-231 (TNBC) versus MCF-10A cells based on the average Normalized Area of each protein entry revealed promising molecules to investigate further as potential specific biomarkers or drug targets for HER2+ BC or TNBC. To narrow down the list of candidate targets in HER2+ BC and TNBC the following criteria were applied:

(1) Candidate proteins were only detected in the cancerous cell lines (HCC-1954 and MDA-MB-231) compared to the normal control cell line (MCF-10A).
(2) Protein expression levels in the normal cell line were defined as those proteins that were identified in at least three replicates with a Normalized Area ≥ 70; Candidate proteins were highly abundant. The proteins with the highest Normalized Area were reported.
(3) The percent Coefficient of Variation (% CV) between replicates of each candidate protein was ≤ 40, based on statistical analysis. Applying these criteria led to the selection of the top 30 candidate protein targets in HER2+ BC and TNBC as shown in **Tables 1** and **2**, respectively.

**Table 1:** Selected membrane associated proteins specifically identified in HCC-1954 compared to MCF-10A cells.

Uniprot accession no.	Gene names	Protein names	Average Normalized_Area	SD
P04626	ERBB2	Receptor tyrosine-protein kinase erbB-2	2103	225
Q687X5	STEAP4	Metalloreductase STEAP4	1214	176
Q96KA5	CLPTM1L	Cleft lip and palate transmembrane protein 1-like protein	638	92
P05534	HLA-A	HLA class I histocompatibility antigen, A-24 alpha chain	573	104
Q14451	GRB7	Growth factor receptor-bound protein 7	537	137
Q04826	HLA-B	HLA class I histocompatibility antigen, B-40 alpha chain	442	116
P11166	SLC2A1	Solute carrier family 2, facilitated glucose transporter member 1	433	65
Q14849	STARD3	StAR-related lipid transfer protein 3	403	81
Q03518	TAP1	Antigen peptide transporter 1	376	130
P30504	HLA-C	HLA class I histocompatibility antigen, Cw-4 alpha chain	374	93
O15533	TAPBP	Tapasin	359	107
Q03519	TAP2	Antigen peptide transporter 2	292	47
O95832	CLDN1	Claudin-1	249	74
P49788	RARRES1	Retinoic acid receptor responder protein 1	245	20
P43003	SLC1A3	Excitatory amino acid transporter 1	212	30
P48651	PTDSS1	Phosphatidylserine synthase 1	208	48
Q9NZ08	ERAP1	Endoplasmic reticulum aminopeptidase 1	199	3
Q6PI78	TMEM65	Transmembrane protein 65	199	20
Q8N5K1	CISD2	CDGSH iron-sulfur domain-containing protein 2	186	28
Q9P0I2	EMC3	ER membrane protein complex subunit 3	184	43
Q15392	DHCR24	Delta(24)-sterol reductase	182	16
Q8WY22	BRI3BP	BRI3-binding protein	166	22
Q96HV5	TMEM41A	Transmembrane protein 41A	165	16
Q8NHH9	ATL2	Atlastin-2	164	26
P21397	MAOA	Amine oxidase [flavin-containing] A	162	39
Q92575	UBXN4	UBX domain-containing protein 4	159	39
P53621	COPA	Coatomer subunit alpha	156	33
Q6NUQ4	TMEM214	Transmembrane protein 214	156	24
P29317	EPHA2	Ephrin type-A receptor 2	153	18
Q9BPW9	DHRS9	Dehydrogenase/reductase SDR family member 9	153	44

**Table 2:** Selected membrane associated proteins specifically identified in MDA-MB-231 compared to MCF-10A cells.

Uniprot accession no.	Gene names	Protein names	Average Normalized_Area	SD
P01892	HLA-A	HLA class I histocompatibility antigen, A-2 alpha chain	4575	1130
P10316	HLA-A	HLA class I histocompatibility antigen, A-69 alpha chain	4409	1138
Q95604	HLA-C	HLA class I histocompatibility antigen, Cw-17 alpha chain	4114	1126
P30479	HLA-B	HLA class I histocompatibility antigen, B-41 alpha chain	1382	367
Q9NZ08	ERAP1	Endoplasmic reticulum aminopeptidase 1	473	73
P42892	ECE1	Endothelin-converting enzyme 1	443	78
P04233	CD74	HLA class II histocompatibility antigen gamma chain	412	162
P29966	MARCKS	Myristoylated alanine-rich C-kinase substrate	303	122
P29317	EPHA2	Ephrin type-A receptor 2	276	98
Q03518	TAP1	Antigen peptide transporter 1	267	76
P48960	CD97	CD97 antigen	262	72
P13760	HLA-DRB1	HLA class II histocompatibility antigen, DRB1-4 beta chain	246	49
O75976	CPD	Carboxypeptidase D	233	54
Q9NZB2	FAM120A	Constitutive coactivator of PPAR-gamma-like protein 1	226	78
P35610	SOAT1	Sterol O-acyltransferase 1	215	29
Q9NRX5	SERINC1	Serine incorporator 1	210	80
P11717	IGF2R	Cation-independent mannose-6-phosphate receptor	198	43
P01903	HLA-DRA	HLA class II histocompatibility antigen, DR alpha chain	194	30
P30530	AXL	Tyrosine-protein kinase receptor UFO	191	50
Q9BYC5	FUT8	Alpha-(1,6)-fucosyltransferase	180	45
Q15599	SLC9A3R2	Na(+)/H(+) exchange regulatory cofactor NHE-RF2	174	26
P01130	LDLR	Low-density lipoprotein receptor	171	63
Q9ULC5	ACSL5	Long-chain-fatty-acid–CoA ligase 5	170	32
P60059	SEC61G	Protein transport protein Sec61 subunit gamma	165	53
P08174	CD55	Complement decay-accelerating factor	165	28
Q9Y371	SH3GLB1	Endophilin-B1	165	49
P13498	CYBA	Cytochrome b-245 light chain	164	74
Q15382	RHEB	GTP-binding protein Rheb	161	50
Q14739	LBR	Lamin-B receptor	160	63
P34810	CD68	Macrosialin	158	29

Notably, among the list of identified candidates exclusively found in the HER2+ BC cell line (**Table 1**) the most abundant was the driver proto-oncogene and key biomarker that characterizes this type of cancer, the human epidermal growth factor receptor 2 (ERBB2, also known as HER2) (31), verifying the reliability and validity of the described proteomic methodology. Furthermore, several well-established HER2+ BC proteins as well as novel BC protein targets were specifically identified in the HER2+ BC cell line (**Table 1**). Among the proteins previously known to be up-regulated in HER2+ BC, were GRB7, STARD3 and EPHA2. Specifically, the adapter protein, GRB7, and the cholesterol-binding protein, STARD3, have been shown to be coamplified and co-overexpressed as part of HER2/ERBB2 17q12 amplicon in HER2+ BC and thus they have been suggested to promote proliferation and contribute to the aggressive behaviour of HER2+ BC (32–35). They have also been implicated in resistance to trastuzumab and Lapatinib treatment (35, 36). Of note, the receptor tyrosine kinase EPHA2 has been involved in breast tumor initiation and metastatic progression of HER2+ BC by amplifying HER2 signalling (37, 38). Furthermore, EPHA2 overexpression in HER2+ BC cells has been demonstrated to confer innate resistance to trastuzumab, indicating that it could be used as a drug target or prognostic marker for patients with trastuzumab-resistant HER2+ BC (39, 40). The above findings constitute confirmation of our methodological approach. Interestingly, some novel BC related proteins (STEAP4, CLPTM1L, TMEM41A, DHCR24, etc., shown in **Table 1**) were also discovered, which have been reported to be involved in other cancer types. Among the novel BC proteins identified, the six-transmembrane epithelial antigen of the prostate 4 (STEAP4) drew our strong interest due to its key role in iron and copper homeostasis and previous reports in prostate and colon cancer (41–43). Due to its membrane-bound localization and its high expression in prostate and colon cancer, STEAP4 has been suggested as a promising therapeutic target (42, 43).

With respect to TNBC, among the list of identified candidates specifically found in the TNBC cell line (**Table 2**), there were some proteins (CD74, EPHA2, CD97, AXL and LDLR) previously known to be overexpressed or implicated in TNBC. More specifically, the overexpression of the transmembrane glycoprotein CD74 has been significantly correlated with TNBC and lymph node metastasis (44, 45). The receptor tyrosine kinase EPHA2 overexpression has been found in the basal-like molecular subtype of BC and has been proposed to promote tumor invasiveness in TNBC and correlate with poor recurrence-free survival (RFS) in TNBC (46, 47). Remarkably, the G protein-coupled receptor (GPCR), CD97, was found for the first time in TNBC by a global proteomic profiling approach, confirming the potential of our proteomic strategy in identification of low abundant molecules with challenging detection, such as GPCRs. However, its implication in TNBC has been previously reported in some studies (48, 49). CD97 is the most broadly expressed member of the epidermal growth factor seven-span transmembrane (EGF-TM7) subfamily of adhesion GPCRs, that is involved in cell proliferation, differentiation, and apoptosis (50). CD97 has been demonstrated to confer an invasive phenotype and its interaction with its ligand, CD55, has been shown to contribute to tumorigenesis through cell adhesion, migration, and angiogenesis through G-protein dependent or G-protein-independent signaling (50, 51). The receptor tyrosine kinase AXL has been demonstrated to be preferentially upregulated in TNBC and its high expression levels has been associated with reduced RFS and OS in TNBC patients treated with anthracycline–taxane-based adjuvant chemotherapy (52, 53). Moreover, the elevated expression of the low density lipoprotein receptor (LDLR) in TNBC cells has been demonstrated to be consistent with the aggressive and metastatic nature of TNBC (54, 55). Four candidate membrane proteins from the lists of specifically detected proteins in HER2+ BC and TNBC cell lines (**Tables 1**, **2**) were chosen for further verification of the proteomic approach, based on their implication in cancer, their significance in each subtype of BC, their identified high expression levels and their novelty. Among them, were two well-established BC receptors (HER2 and EPHA2), a previously studied GPCR in TNBC (CD97) and a novel BC involved metalloreductase STEAP4. Remarkably, CD97 and STEAP4 emerged as novel candidate markers for TNBC and HER2+ BC, respectively. CD97 expression levels have been previously reported in a TNBC cell line (48, 49), but never before by a global proteomic technology. Regarding STEAP4, to our knowledge this is the first time that its up-regulation is associated with BC, and more specifically with HER2+ BC.

### Verification of Selected Candidate Protein Targets *via* Western Blot and IF Analysis

**Figure 3:**
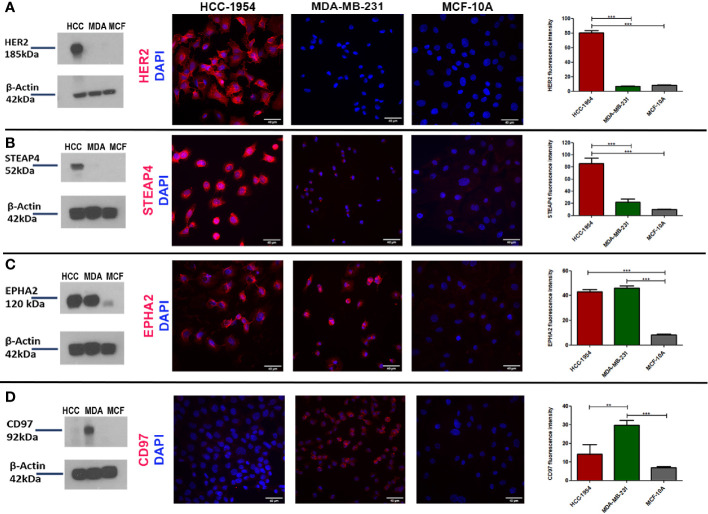
Verification of the expression pattern of the selected protein targets identified by GeLC-MS/MS with Western blot and Immunofluorescence (IF) analysis. **(A, B)** Consistent with the proteomic results, Western blot detection of HER2 and STEAP4 in BC cell lines was only found in HCC-1954 (HER2+) cells. IF qualitative and relative quantitative results of HER2 and STEAP4 confirmed their overexpression in HCC-1954 cells as compared to the control MCF-10A and the MDA-MB-231 (TNBC) cells (***p < 0.001). **(C)** EPHA2 was highly expressed in MDA-MB-231 and HCC-1954 but faintly detectable in the control MCF-10A cells by Western Blot and IF qualitative and quantitative analysis, as we expected from the proteomic results (***p < 0.001). **(D)** CD97 expression was only detected in MDA-MB-231 (TNBC) through Western blot and IF results (**p < 0.01, ***p < 0.001). Scale bars in the IF images represent 40 µm.

To further verify the expression pattern of the four selected candidate membrane protein targets, Western blot and Immunofluoresence (IF) analysis of HER2, STEAP4, EPHA2 and CD97 were performed from the whole cell lysates of the HCC-1954, the MDA-MB-231 and the MCF-10A cell lines. Results are depicted in **Figure 3**. The Western blot analysis was in good agreement with the proteomic results, since HER2 and STEAP4 expression was detected only in the HCC-1954 cell line (**Figures 3A, B**), CD97 was identified only in the MDA-MB-231 cell line (**Figure 3D**) and EPHA2 was solely increased in the HCC-1954 and the MDA-MB-231 cell lines compared to a low expression in the MCF-10A cell line (**Figure 3C**). The whole uncropped images of the original Western blots for **Figure 3** are provided in **Supplementary Figure 4**. IF analysis further confirmed the proteomic data, Protein specific IF staining demonstrated that HER2 and STEAP4 expression was upregulated in the HCC-1954 cell line compared to the MDA-MB-231 and the MCF-10A cell lines (**Figures 3A, B**), whereas CD97 was predominantly present in the MDA-MB-231 cell line (**Figure 3D**). Finally, IF staining of EPHA2 showed that it was abundantly identified in the HCC-1954 and the MDA-MB-231 compared to almost no expression in the MCF-10A cell line (**Figure 3C**). Further validation studies by Western blot and IF analysis of STEAP4, confirmed its expression in other HER2+ BC cell lines, SKBR3 and BT474 (**Supplementary Figure 1**).

In conclusion, the differential expression levels of these proteins confirmed the findings derived from the proteomic data analysis. While the presence of the adhesion GPCR CD97 has been previously described in BC, the mitochondrial iron reductase STEAP4, to the best of our knowledge, has not been previously implicated in BC (HER2+ subtype). Thus, the novelty and abundance of STEAP4 prompted us to select it for further evaluation and pharmacological analysis.

### Tissue Array Profiling of STEAP4 Expression Association With Clinicopathological Parameters

To assess the clinical relevance of STEAP4 in BC and normal breast tissues, IHC analysis of STEAP4 was conducted initially on tissues derived from mice inoculated with the HCC-1954 and MDA-MB-231 cell lines and on two independent BC TMAs (BRC253, BRC961) containing a total of 18 benign and 42 malignant breast tissues.

**Figure 4:**
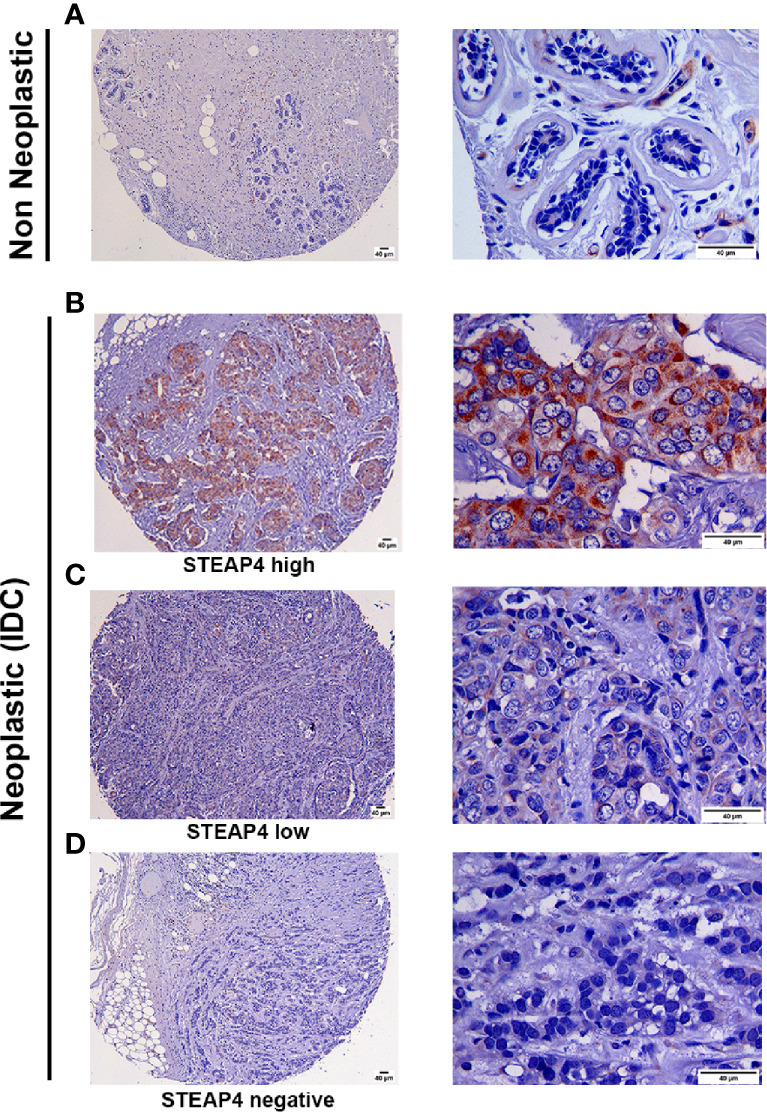
Immunohistochemical analysis of STEAP4 expression in human BC tissues. STEAP4 expression was assessed in 2 independent tissue microarray (TMA) slides, containing 6 cases of invasive ductal carcinoma and adjacent normal breast tissue (BR251c) and 36 cases of breast cancers and 12 cases of normal and benign breast tissues (BRC961). Representative IHC images (100x and 400x magnifications, scale bars = 40 μm) of **(A)** negative STEAP4 expression in a non-neoplastic breast tissue, **(B)** high STEAP4 expression, in an invasive ductal carcinoma, **(C)** low STEAP4 expression in an invasive ductal carcinoma and **(D)** negative STEAP4 expression in an invasive ductal carcinoma from the TMAs are depicted.

Consistent with Western blot and IF results, the HCC-1954 xenograft tissues were positive for STEAP4, whereas the MDA-MB-231 xenograft tissues were negative as shown in **Supplementary Figure 2**. Representative IHC images of varying levels of STEAP4 expression in the TMAs of non-neoplastic and neoplastic breast tissues are depicted in **Figure 4**. STEAP4 showed a cytoplasmic and focally paranuclear staining pattern in both xenografts and TMAs; however, a plasma membrane localization was also detected in our IF investigation, which is in concordance with previous studies (41, 43).

Association between STEAP4 expression and clinicopathological features derived from the two TMAs are merged and summarized in **Table 3**. The data were expressed in terms of STEAP4 positive versus negative cases. Notably, STEAP4 expression was found only in malignant breast tissues whereas all the benign breast cases/tissues had no detectable levels of STEAP4 (*p* < 0.01). In addition, 49% (20/41) of the BC tissues showed positive STEAP4 expression while 51% (21/41) of the BC cases were STEAP4 negative (**Table 3**). STEAP4 expression was significantly associated with the tumor grade (*p* < 0.01). However, no statistically significant correlations were observed between STEAP4 expression and all the other clinicopathological features, although a strong trend was evident with lymph node metastasis and a weaker one with pT status. These results clearly indicated that STEAP4 is involved in the pathogenesis of human BC, however possibly due to the small number of the clinical samples we couldn’t determine significant associations between STEAP4 and specific clinicopathological parameters such as HER2 status, age, pT status, histology grade, lymph node metastasis or pathological stage.

**Table 3:** Association of STEAP4 expression with patient’s clinicopathological features in the TMA slides.

Clinical pathological parameters	Case number	STEAP4 positive	STEAP4 negative	P-values
**Normal & Benign vs Cancer**				
Normal & Benign	17/18	0	17	**0.0004**
Cancer	41/42	20	21	
**CANCER CASES**				
**Age (years)**				
> 40	24/42	14	10	0.1459
≤ 40	17/42	6	11	
**pT status**				
T2	24/42	15	9	0.0836
T3	10/42	3	7	
**Histology grade**				
II	10/42	10	0	**0.0051**
II~III	8/42	4	4	
III	12/42	4	8	
**Lymph node metastasis**				
Negative	20/42	7	13	0.0578
Positive	20/42	13	7	
**Stage**				
IIA	10/42	5	5	0.3729
IIB	20/42	9	11	
IIIA	5/42	4	1	
**HER2 status**				
Negative	18/42	7	11	0.8902
Positive	17/42	7	10	

Chi-square test was used to test statistical association. Statistical significant *P*-values are shown in bold.

Remarkably, due to its membrane-bound localization and its high over-expression in neoplastic compared to non-neoplastic tissues, STEAP4 constitutes an ideal pharmacological target for cancer therapy.

### Synergistic Efficacy Through Combination of HER2 and STEAP4 Inhibition in HER2+ BC

Based on our findings, STEAP4 could be used as a novel candidate therapeutic target for HER2+ BC. A recent study has shown that an FDA-approved iron chelator, Deferiprone (DFP), can inhibit the STEAP4 pathway (42). Therefore, in order to evaluate the pharmacological relevance of STEAP4 in HER2+ BC, a combination of DFP with a typically used HER2 inhibitor, Lapatinib, was employed for the testing treatment of a STEAP4+ and a STEAP4- BC cell lines. We hypothesized that these two drugs could have a synergistic effect on the growth of the HER2+ BC cells only, and could ultimately improve the efficacy and tolerability of Lapatinib therapy due to resistance issues.

**Figure 5:**
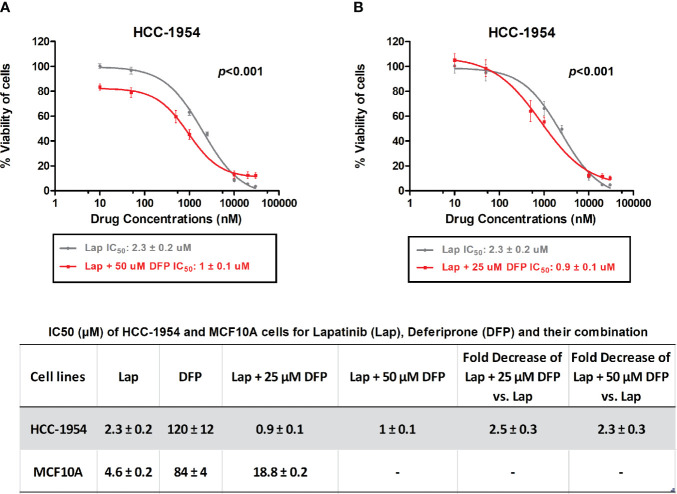
*In vitro* MTT cytotoxicity of Lapatinib compared to combined treatment of Lapatinib and Deferiprone in HCC-1954 (HER2+) cells. **(A)** A synergistic cell growth inhibitory effect of Lapatinib in combination with 50 μM Deferiprone on HCC-1954 cells was found (*p* < 0.001). **(B)** A similar synergistic cell growth inhibitory effect was determined for Lapatinib in combination with 25 μM Deferiprone on HCC-1954 cells (*p* < 0.001). The results represent the mean values ± SD of three independent experiments.

Given the fact that Lapatinib is a dual EGFR/HER2 inhibitor, we chose the HER2 overexpressing BC cell line, HCC-1954, and the EGFR overexpressing benign control cell line, MCF-10A, for further evaluation. Moreover, STEAP4 expression levels were highly and consistently detectable in the HCC-1954 cell line whereas the MCF-10A cell line had undetectable levels of expression, based on our results. We therefore studied the sensitivity of the selected BC cell lines to Lapatinib and DFP, either alone or in combination after a 72-hour treatment. As shown in **Figures 5A, B**, Lapatinib alone produced a gradual dose-dependent growth inhibition in both the HCC-1954 and the MCF-10A cell lines whereas DFP alone had very limited cytotoxic effect. More specifically, after 72-h exposure to Lapatinib, the IC_50_ values in the HCC-1954 and the MCF-10A cells were 2.3 ± 0.2 and 4.6 ± 0.2 μM, respectively, whereas after DFP treatment, the IC_50_ values in the HCC-1954 and the MCF-10A, were 120 ± 12 and 84 ± 4 μM, respectively. Importantly, when cells were exposed to increasing doses of Lapatinib (10 nM-30 μM) in combination with DFP at 25 μM or 50 μM, synergistic activity was observed only in the HCC-1954 cells, resulting in a ~60% growth-inhibition, with an IC_50_ of approximately 1 μM (*p* < 0.001). Given the fact that the growth of the MCF-10A cells was not affected by the combination treatment with Lapatinib and DFP, we concluded that the additive growth inhibitory effect in the HER2+ cell line, HCC-1954, could also be mediated through the STEAP4 pathway inhibition. A summary of the IC_50_ values of Lapatinib and DFP, either alone or in combination is provided. A similar synergistic cell growth inhibitory effect (~50%) was also observed for Lapatinib in combination with 25 μM Deferiprone in the other tested HER2+ BC cells, SKBR3 and BT474 (*p* < 0.001), as shown in **Supplementary Figure 3**. It should be noted that Lapatinib sensitivity of the SKBR3 and the BT474 cells was much higher than that of the HCC-1954 cells, a finding that is consistent with the literature (56–58).

### Knockdown of STEAP4 Suppressed Cell Proliferation and the IC50 of Lapatinib in HCC-1954 Cells

**Figure 6:**
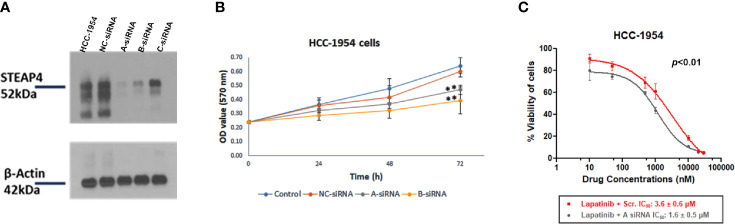
Effect of STEAP4 silencing on cell proliferation and Lapatinib inhibition in HCC-1954 (HER2+) cells. **(A)** Knockdown efficiency of the three siRNA duplexes on STEAP4 protein expression in HCC-1954 cells, as assessed by Western blot analysis 48 h after transfection with 10nM siRNAs. **(B)** HCC-1954 cell growth curves at 24, 48, and 72 h after transfection with 10nM siRNAs. Cell proliferation OD values were detected by MTT assay. STEAP4 A-siRNA and B-siRNA significantly reduced cell proliferation at 72 h of transfection as compared to the NC-siRNA (non-silencing of STEAP4) or Control group (**p < 0.01). **(C)** Dose–response curves and the IC50 values after siRNA and Lapatinib treatment. HCC-1954 cells were treated with a fixed concentration of siRNAs (10 nM) and different concentrations of Lapatinib for 72 h to assess the effect of A-siRNA and Lapatinib on cell viability using the MTT assay. NC-siRNA (non-silencing of STEAP4) was used as the negative control for comparison (*p* < 0.01). The results represent the mean values ± SD of three independent experiments.

To examine the silencing efficiency of siRNAs on STEAP4 protein, three siRNA duplexes (A-siRNA, B-siRNA and C-siRNA) and a negative control (NC-siRNA), which is absent in human genomes, were used for transfection of the HCC-1954 cells. The effects of the specific siRNAs on STEAP4 protein expression were evaluated by Western blot analysis, after the treatment of 10nM for each siRNA. The results indicated that STEAP4 expression was significantly decreased in the A-siRNA and B-siRNA treated groups compared with the C-siRNA, NC-siRNA treated groups and untreated group (**Figure 6A**). Transfection with NC-siRNA did not alter STEAP4 expression levels, indicating that the inhibitory effect of STEAP4 siRNA was specific. Taken together, the A-siRNA and B-siRNA were considered as optimal interference sequences, which were used for subsequent experiments.

After A-siRNA, B-siRNA and NC-siRNA of STEAP4 were transfected into HCC-1954 cells for 14 h, the cells were cultured for 24, 48, and 72 h and then treated with the MTT solution. The NC-siRNA was used as a negative control to compare nonspecific toxicity. Absorbance in the cells was detected respectively after which the cell growth curve was drawn (**Figure 6B**). The cell growth in the A-siRNA, and B-siRNA groups at 24 and 48 h was not significantly affected when compared with the NC-siRNA and the Control (mock transfected) groups. However, cell proliferation at 72 h was significantly inhibited (*p* < 0.01) in the A-siRNA and B-siRNA groups.

Since A-siRNA had the most marked silencing effect among the three siRNAs assessed (**Figure 6A**), it was selected for further pharmacological evaluation.

To investigate the effect of siRNA-A treatment on the chemosensitivity of HCC-1954 cells to Lapatinib treatment, cells were treated with 10 nM of A-siRNA or NC-siRNA for 14 h followed by treatment of different concentrations of Lapatinib. The IC_50_ values of Lapatinib following transfection were determined (**Figure 6C**). After 72 h exposure to increasing doses of Lapatinib (10 nM-30 μM), the IC_50_ values in the A-siRNA transfected HCC-1954 cells were 1.6 ± 0.5 μM, whereas the IC_50_ values in the NC-siRNA transfected were 3.6 ± 0.6 μM. These results indicated a statistical significant increase in the Lapatinib inhibition of the HCC-1954 cells when treated with A-siRNA compared with the control group (*p* < 0.01).

## Discussion

Our study was not limited to presenting a wide dynamic range, sensitive and robust approach for BC proteomic analysis, but it also advanced our understanding on the proteomic profile of BC with the discovery of potential biomarkers and therapeutic candidate targets for pharmacological intervention. The combination of a membrane enrichment protocol with the label-free GeLC-MS/MS quantitative proteomic profiling generated a total of 536, 641 and 604 reliable membrane-associated proteins from the HCC-1954, the MDA-MB-231 and the MCF-10A cell lines. Deep-dive proteomic data analysis showed that 110 and 191 proteins were exclusively present in the HCC-1954 and the MDA-MB-231 cell lines, respectively compared to the MCF-10A cell line. Several of the proteins identified in our study have been reported previously to be elevated in HER2+ BC and TNBC, suggesting that the GeLC-MS/MS methodology is a powerful screening tool for the identification of novel biomarkers for aggressive subtypes of BC. To our knowledge, this is the first time that the six-transmembrane epithelial antigen of prostate 4 (STEAP4) which emerged from our screening efforts is linked to BC, and provides a novel pharmacological target with potential clinical translation.

STEAP4 is an iron-metabolism-related mitochondrial protein that functions as a metalloreductase involved in cellular iron and coper homeostasis and its expression has been modulated in response to inflammation, oxidative stress and metabolism of fatty acids and glucose (41). Regarding its crucial role in carcinogenesis, STEAP4 overexpression has been previously described in human colon cancer (CRC) and androgen-dependent prostate cancer (PCa) compared to normal colon and prostate (42, 59). Additionally, STEAP4 has been shown to increase reactive oxygen species (ROS) in PCa cells through its iron reductase activity, which may promote PCa tumorigenesis and progression. Mitochondrial iron dysfunction *via* STEAP4 has also been implicated in increased oxidative stress in colon tissues and as a result promoting inflammatory tissue injury and colitis-associated colon cancer. The localization of STEAP4 in the mitochondria and differential expression in normal and cancer tissue make STEAP4 a potential candidate as a biomarker or a therapeutic target in CRC and androgen-dependent PCa.

The overexpression of STEAP4 in the HER2+ BC cells which emerged for the first time in our screening efforts, in tandem with the IHC results in the TMAs, was the inflection point which triggered us to proceed to the pharmacological evaluation of the STEAP4 in HER2+ BC models.

A frequently used iron chelating drug, DFP, has been recently demonstrated to inhibit STEAP4 and as a result it could be used as a promising pharmacological regime against various types of cancer where STEAP4 overexpression is observed. Iron chelators have been previously explored as cancer chemopreventive and chemotherapeutic agents (60). DFP is a well-tolerated orally administered iron chelator, used clinically, which has shown great patient compliance and efficacy in the removal of potentially toxic excess iron from the heart (60, 61) and tissues (62, 63). DFP has been shown to inhibit cancer cell growth through a variety of mechanisms such as involving inhibition of iron-dependent translational and enzymatic processes (61, 62) and molecules, such as STEAP4 (42).

With respect to HER2+ BC, there have been significant therapeutic advances in the field over the last decade, with several potent targeted treatments clinically available, including trastuzumab and Lapatinib alone or in combination with chemotherapeutics and new agents targeting other related to HER2 pathways (6, 64). Lapatinib, the small molecule tyrosine kinase inhibitor which targets HER2 and EGFR, has considerable anti-tumor activity against HER2+ BC cells, including trastuzumab resistant cells. It has also an acceptable safety profile for the treatment of HER2+ BC and unlike trastuzumab, has less cardiotoxicity and may penetrate the blood brain barrier better in the context of CNS metastases (65, 66). However, Lapatinib treatment is associated with hepatobiliary toxicities and frequent disease recurrence due to either innate or acquired drug resistance (67). Therefore, simultaneous pharmacological targeting of crucial metabolic pathways with a combination of targeted agents, as in other types of co-administration clinical models, could prevent or delay the onset of resistance phenomena and possibly contribute to liver safety events in HER2+ BC patients. In this context, we hypothesized that pharmacological blockage of the STEAP4 pathway with the iron chelator DFP in combination with Lapatinib may improve efficacy and/or overcome drug resistance in HER2+ BC.

Consistent with our hypothesis, the dual drug inhibition of HER2 and STEAP4 by the combinational treatment of Lapatinib with the DFP significantly decreased cell proliferation in the HER2+ BC cell lines (HCC-1954, SKBR3, BT474) than either drug alone, suggesting a new pharmacological treatment scheme for HER2+ BC. Future preclinical studies on the appropriate dose adjustment during this combinational treatment may be needed to minimize untoward Lapatinib-related hepatotoxicities. Furthermore, silencing of STEAP4 using small interfering RNA also resulted in a significant reduction in cell growth and enhanced the inhibition potential of the Lapatinib in the HCC-1954 cells.

Based on our pharmacological findings *via* the combinatorial treatment with the iron chelator, DFP, and lapatinib, and given STEAP4’s role in intracellular iron import, we hypothesize that the mitochondrial iron pathway plays a potentially critical and understudied role in the pathogenesis and/or progression of HER2+ BC. Supporting evidence of this is provided by studies that correlate HER2 overexpression and elevated iron levels (68, 69). Evidence in the literature associates STEAP4 with metabolic dysregulations in cancer and other diseases, where inflammation is a central hallmark. Inflammatory cytokines like IL17, have been linked with STEAP4 upregulation and cancer progression (70). Other key proinflammatory cytokines like TNFα, IL-6 and IL-1β were also associated with STEAP4 overexpression (41). In a similar pattern, the HER2 overexpression has also been shown to consistently activate multiple inflammatory pathways, including the secretion of high levels of IL-6 (71, 72). Muraro et al. demonstrated that increased levels of IL-6 and IL-1β were found in the serum of HER2+ patients compared to HER2 negative patients (73). However, despite these findings we are not aware of a published direct linkage between STEAP4 and HER2, necessitating further research on this topic. We hypothesize that in the inflammatory tumor microenvironment, STEAP4 and HER2+ are somehow interconnected with an unidentified direct or indirect mechanism involving inflammatory cytokines that eventually contributes to increased intracellular iron accumulation, which in turn could lead to enhanced oxidative stress promoting HER2+ BC progression (41, 74). Further proteomic and gene expression profiling studies in lapatinib and/or DFP treated, untreated and STEAP4-silenced HER2+ BC cells need investigation in order to identify key signaling differences, direct interacting partners and pathway enrichment correlating with STEAP4 expression. Given the fact that there are no specific STEAP4 inhibitors, the elucidation of the molecular mechanism by which STEAP4 affects the development of HER2+ BC could highlight novel druggable partners for more selective targeting of STEAP4 in HER2+ BC therapy.

In conclusion, STEAP4 may represent a potential BC related biomarker and a promising new pharmacological target for the treatment of HER2+ BC. Further validation studies involving a larger clinical sample size would be necessary to clarify the association of STEAP4 with clinical parameters and its potential role in the tumorigenesis and treatment of HER2+ BC.

## Data Availability Statement

The datasets presented in this study can be found in online repositories. The names of the repository/repositories and accession number(s) can be found below: ProteomeXchange Consortium *via* the PRIDE partner repository with the dataset identifier PXD021819.

## Ethics Statement

All animal procedures were approved by the Bioethical Committee of BRFAA based on the European Directive 86/609.

## Author Contributions

I-MO designed aspects of the study, performed the experiments, analyzed and interpreted the data, and wrote the manuscript. CT conceived and designed the study, wrote and edited the paper and was the principal investigator of the study. OA prepared the xenograft tumor sections and contributed to the critical revision of the manuscript. AP designed the silencing studies. KV performed the mass spectrometry analysis. ST-B evaluated the clinical tissue microarray samples. OA, AP, ST-B, and KV participated in the manuscript revision and were involved in helpful suggestions and discussions. All authors contributed to the article and approved the submitted version.

## Funding

The research work was supported by the Hellenic Foundation for Research and Innovation (HFRI) and the General Secretariat for Research and Technology (GSRT), under the HFRI PhD Fellowship grant (GA. no. 2026). This work was further supported by the “Infrastructure for Preclinical and early-Phase Clinical Development of Drugs, Therapeutics and Biomedical Devices (EATRIS-GR)” (MIS 5028091), which is implemented under the Action “Reinforcement of the Research and Innovation Infrastructure”, funded by the Operational Programme “Competitiveness, Entrepreneurship and Innovation” (NSRF 2014- 2020) and co-financed by Greece and the European Union (European Regional Development Fund).

## Conflict of Interest

The authors declare that the research was conducted in the absence of any commercial or financial relationships that could be construed as a potential conflict of interest.

## Supplementary Material

Supplementary Figure 1 Verification of the STEAP4 expression by Western blot and Immunofluorescence (IF) analysis in HER2+ BC cells. **(A)** STEAP4 expression levels were detected in SKBR3 and BT474 (HER2+) cells, by Western blot analysis. **(B)** IF results of STEAP4 also confirmed its expression in SKBR3 and BT474 cells. Scale bars in the IF images represent 40 µm.

Supplementary Figure 2 Immunohistochemical analysis of STEAP4 expression in human BC xenograft tissues. STEAP4 expression was examined in the HCC-1954 and MDA-MB-231 xenograft tissues. Representative IHC images (200x magnifications, scale bars = 40 μm) of **(A)** positive STEAP4 expression in the HCC-1954 xenograft tissues and **(B)** negative STEAP4 expression in the MDA-MB-231 xenograft tissues are illustrated.

Supplementary Figure 3 In vitro MTT cytotoxicity of Lapatinib compared to combined treatment of Lapatinib and Deferiprone in HER2+ BC cells. **(A)** A synergistic cell growth inhibitory effect of Lapatinib in combination with 25 μM Deferiprone on SKBR3 cells was found (*p* < 0.001). **(B)** A similar synergistic cell growth inhibitory effect was determined for Lapatinib in combination with 25 μM Deferiprone on BT474 cells (*p* < 0.001). The results represent the mean values ± SD of three independent experiments.

Supplementary Figure 4 Uncropped Western blot images from the main text (provided as a separate PDF file). Pictures depict membranes probed with antibodies as indicated on the blots. Black boxes represent final cropped regions shown in manuscript. All top to bottom images are different exposure from the same membrane. **(A)** Whole, uncropped Western blots of Figure 1A. Samples order in the frames from left to right were: MDA-MB-231 cell lysate fraction (MDA cell), MDA-MB-231 cytosolic fraction (MDA cyt.), MDA-MB-231 membrane fraction (MDA mem.), HCC-1954 cell lysate (HCC cell), HCC-1954 cytosolic fraction (HCC cyt.), HCC-1954 membrane fraction (HCC mem.). **(B–D) **Whole, uncropped Western blots of Figure 3. Three biological replicates were loaded for each sample on the same gel. Samples order from left to right for each blot were: HCC-1954 cell lysate, MDA-MB-231 cell lysate, MCF-10A cell lysate (N=3). **(E)** Whole, uncropped Western blots of Figure 6A. Two biological replicates were loaded for each sample on the same gel. Samples order from left to right for each blot were: HCC-1954 cell lysate, NC-siRNA cell lysate, A-siRNA cell lysate, B-siRNA cell lysate, C-siRNA cell lysate (N=2).

Supplementary Table 1 Complete lists of membrane proteins found in the HCC-1954 cells and MCF-10A cells (provided as a separate excel file). Combined lists of all the membrane-associated proteins found in four replicates of the HCC-1954 cells compared to four replicates of the MCF-10A cells along with their Normalized Areas. Bold letter proteins represent the top 30 candidate protein targets shown in the Table 1 of the main text.

Supplementary Table 2 Complete lists of membrane proteins found in the MDA-MB-231 cells and MCF-10A cells (provided as a separate excel file). Combined lists of all the membrane-associated proteins found in four replicates of the MDA-MB-231 versus the MCF-10A cells along with their Normalized Areas. Bold letter proteins represent the top 30 candidate protein targets shown in the Table 2 of the main text.
