# Supplementary figures and images for: Discovery and Pharmacological Evaluation of STEAP4 as a Novel Target for HER2 Overexpressing Breast Cancer
Source: Front Oncol. 2021 Mar 26;11:608201. doi: 10.3389/fonc.2021.608201 (PMC8034292; doi:10.3389/fonc.2021.608201)

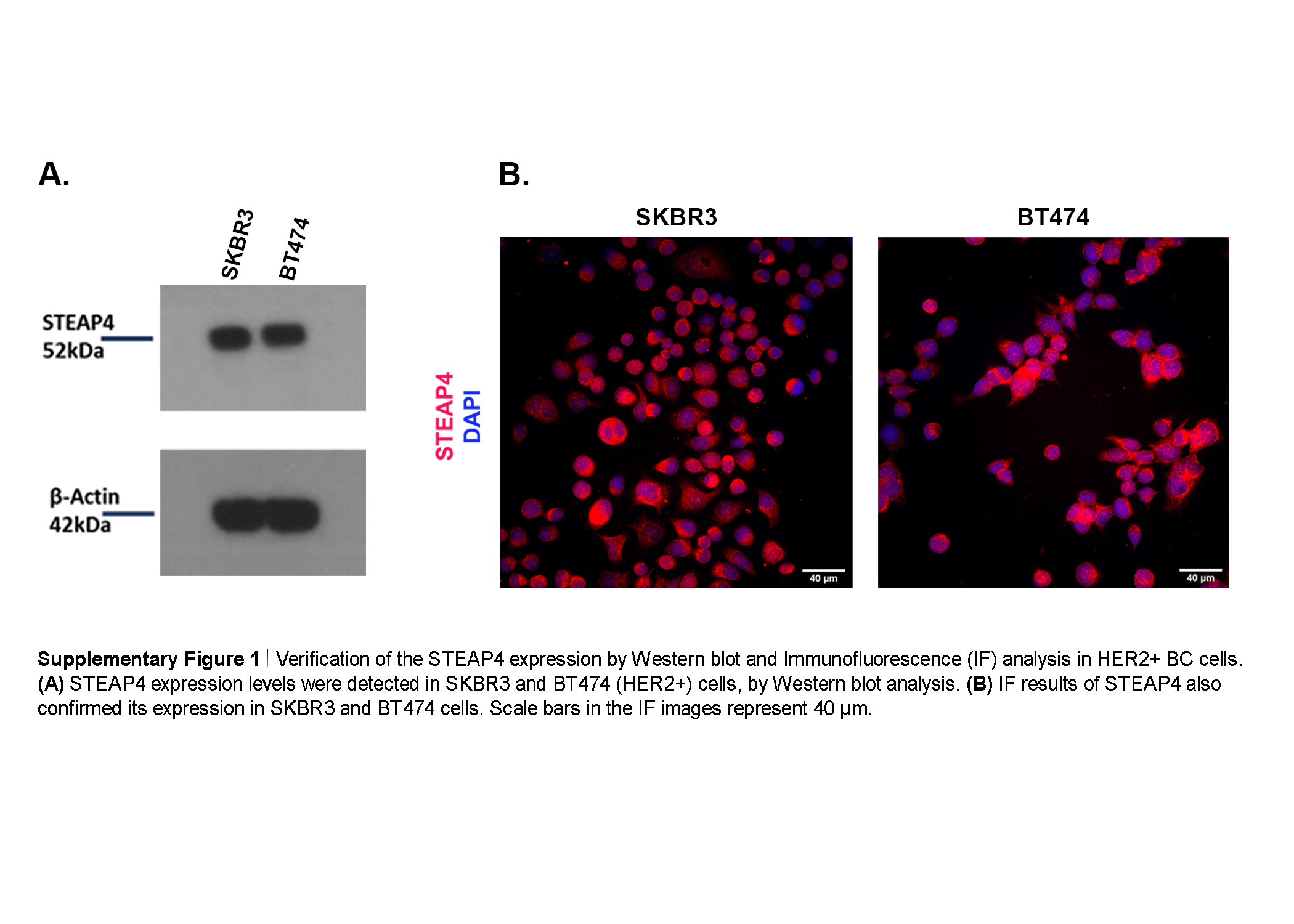

Supplement: Supplementary Figure 1 — Verification of the STEAP4 expression by Western blot and Immunofluorescence (IF) analysis in HER2+ BC cells. (A) STEAP4 expression levels were detected in SKBR3 and BT474 (HER2+) cells, by Western blot analysis. (B) IF results of STEAP4 also confirmed its expression in SKBR3 and BT474 cells. Scale bars in the IF images represent 40 µm. [file Image_1.tif]

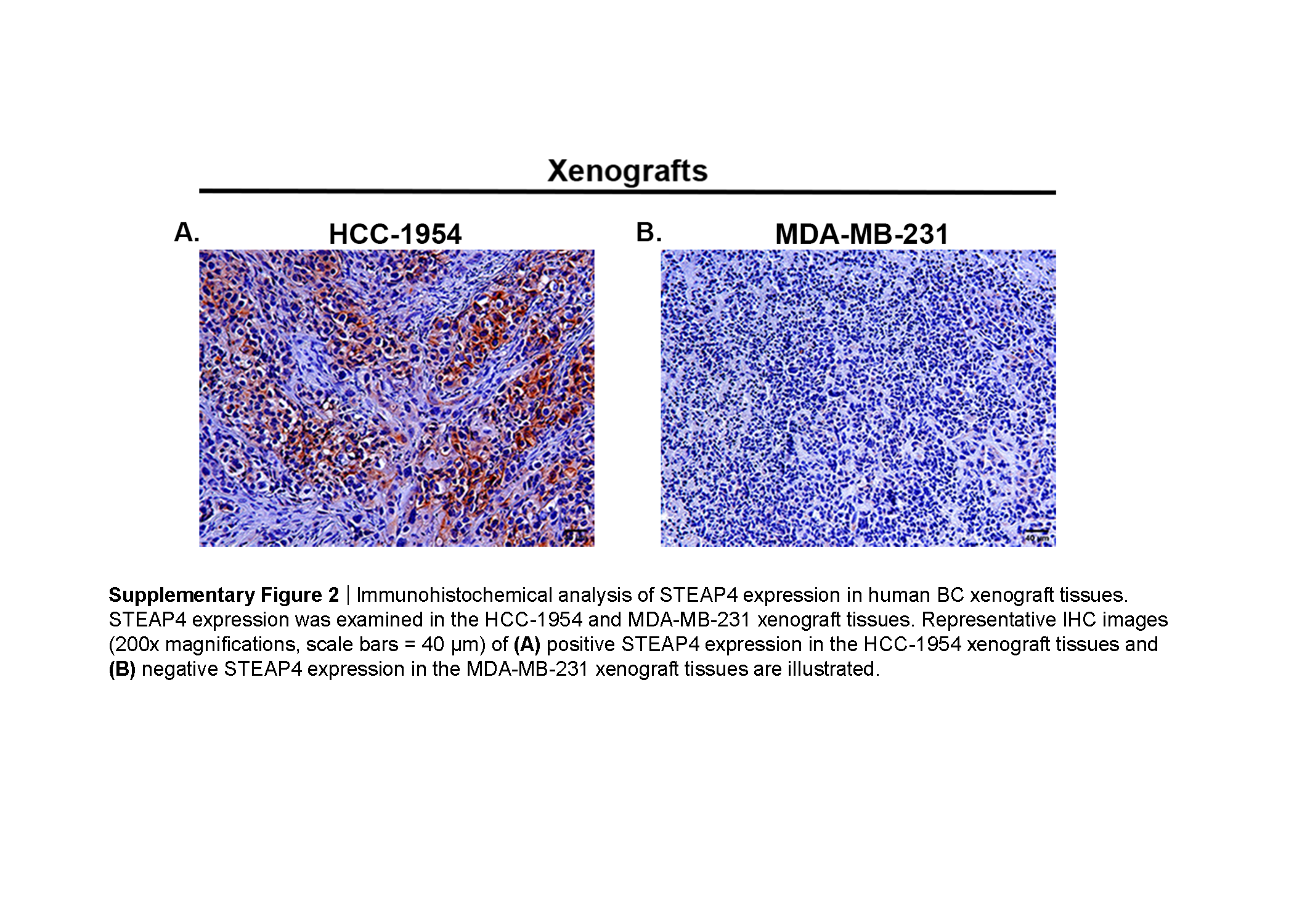

Supplement: Supplementary Figure 2 — Immunohistochemical analysis of STEAP4 expression in human BC xenograft tissues. STEAP4 expression was examined in the HCC-1954 and MDA-MB-231 xenograft tissues. Representative IHC images (200x magnifications, scale bars = 40 μm) of (A) positive STEAP4 expression in the HCC-1954 xenograft tissues and (B) negative STEAP4 expression in the MDA-MB-231 xenograft tissues are illustrated. [file Image_2.tif]

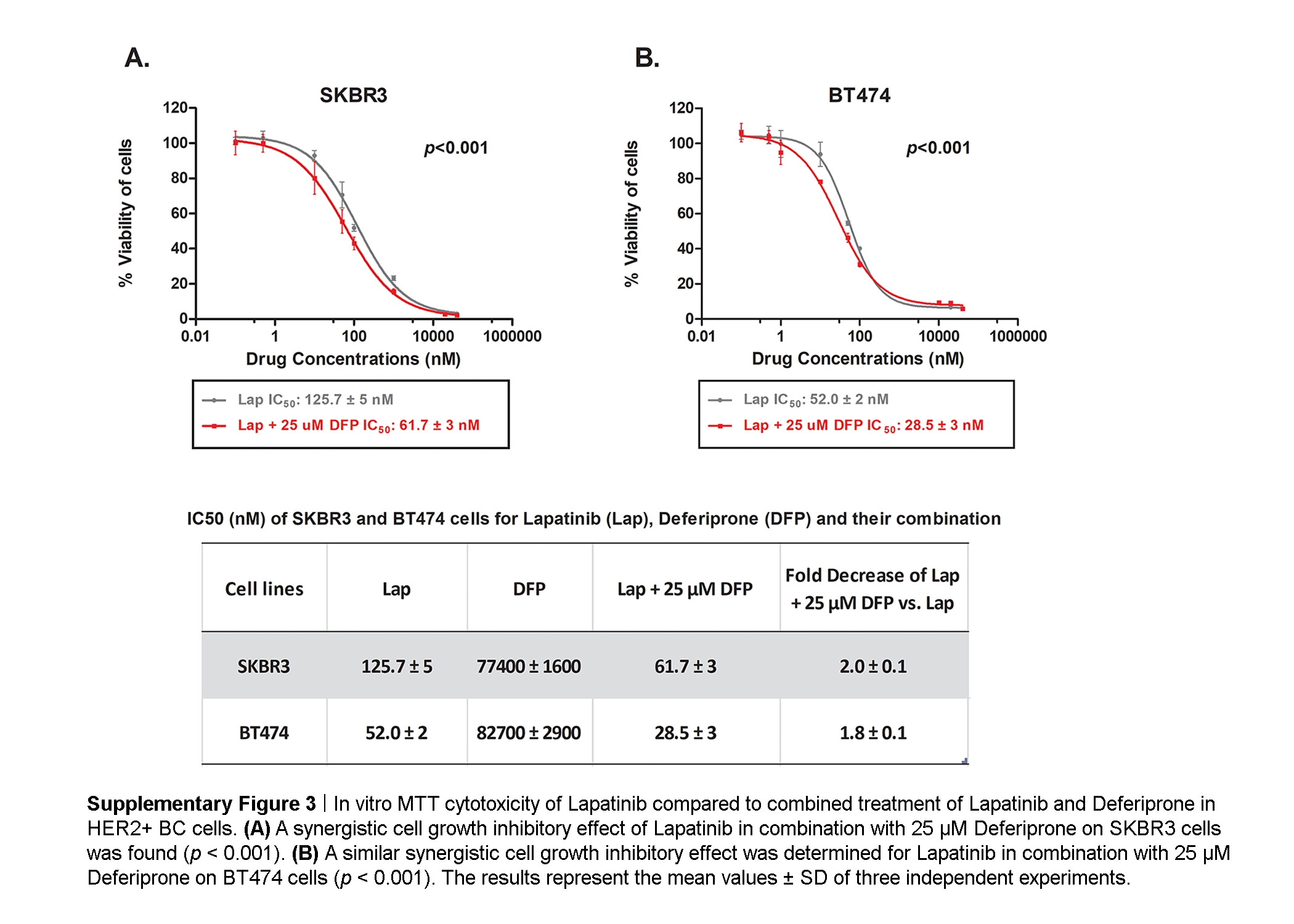

Supplement: Supplementary Figure 3 — In vitro MTT cytotoxicity of Lapatinib compared to combined treatment of Lapatinib and Deferiprone in HER2+ BC cells. (A) A synergistic cell growth inhibitory effect of Lapatinib in combination with 25 μM Deferiprone on SKBR3 cells was found (p < 0.001). (B) A similar synergistic cell growth inhibitory effect was determined for Lapatinib in combination with 25 μM Deferiprone on BT474 cells (p < 0.001). The results represent the mean values ± SD of three independent experiments. [file Image_3.tif]
