# Supplementary material for: Discovery and Pharmacological Evaluation of STEAP4 as a Novel Target for HER2 Overexpressing Breast Cancer
Source: Front Oncol. 2021 Mar 26;11:608201. doi: 10.3389/fonc.2021.608201 (PMC8034292; doi:10.3389/fonc.2021.608201)
Supplement: Supplementary Figure 4 — Uncropped Western blot images from the main text (provided as a separate PDF file). Pictures depict membranes probed with antibodies as indicated on the blots. Black boxes represent final cropped regions shown in manuscript. All top to bottom images are different exposure from the same membrane. (A) Whole, uncropped Western blots of Figure 1A. Samples order in the frames from left to right were: MDA-MB-231 cell lysate fraction (MDA cell), MDA-MB-231 cytosolic fraction (MDA cyt.), MDA-MB-231 membrane fraction (MDA mem.), HCC-1954 cell lysate (HCC cell), HCC-1954 cytosolic fraction (HCC cyt.), HCC-1954 membrane fraction (HCC mem.). (B–D) Whole, uncropped Western blots of Figure 3. Three biological replicates were loaded for each sample on the same gel. Samples order from left to right for each blot were: HCC-1954 cell lysate, MDA-MB-231 cell lysate, MCF-10A cell lysate (N=3). (E) Whole, uncropped Western blots of Figure 6A. Two biological replicates were loaded for each sample on the same gel. Samples order from left to right for each blot were: HCC-1954 cell lysate, NC-siRNA cell lysate, A-siRNA cell lysate, B-siRNA cell lysate, C-siRNA cell lysate (N=2). [file Presentation_1.pptx]

## Slide 1
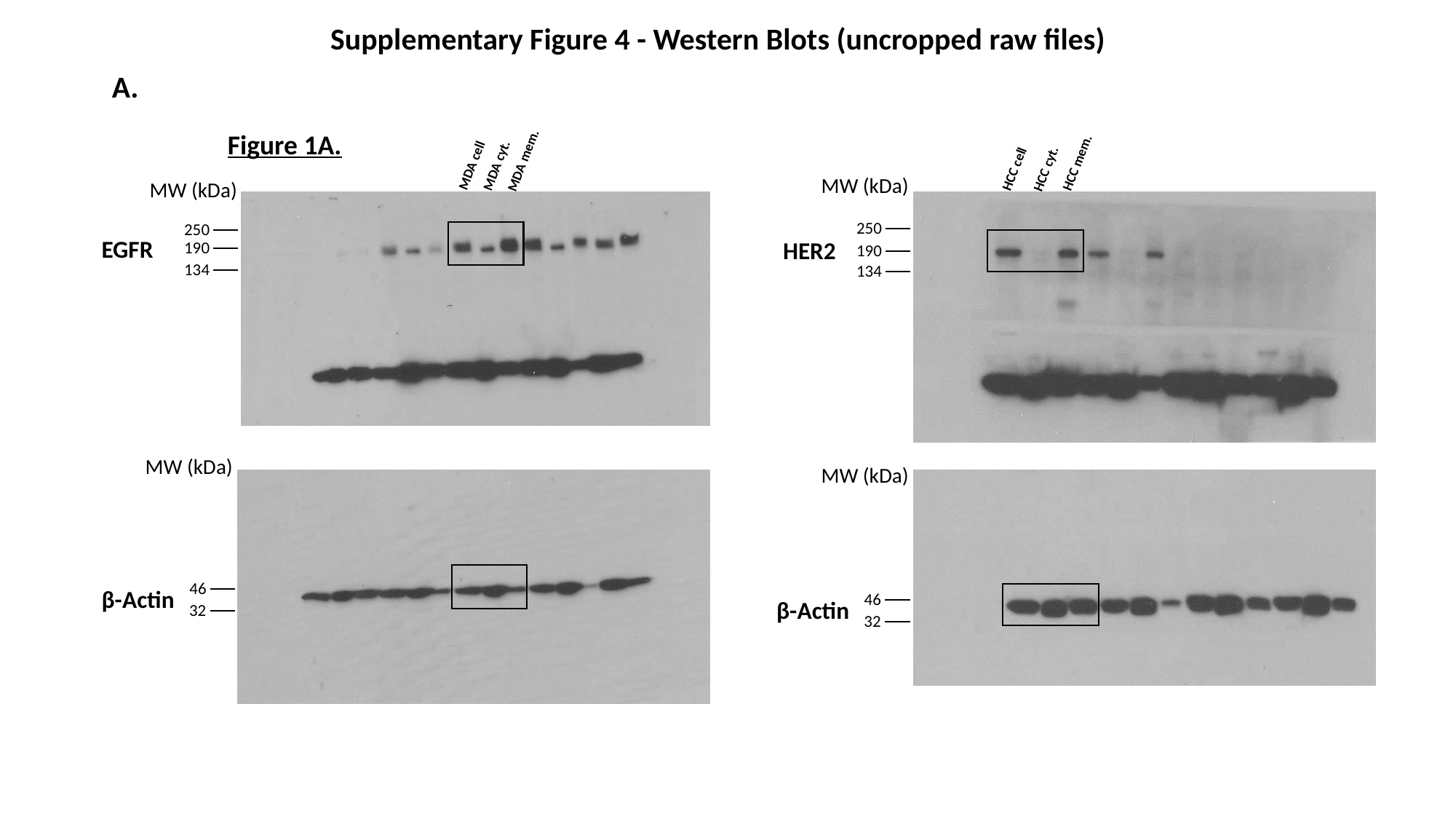

Supplementary Figure 4 - Western Blots (uncropped raw files)
A.
MDA mem.
MDA cyt.
MDA cell
HCC mem.
HCC cyt.
HCC cell
Figure 1A.
MW (kDa)
MW (kDa)
250
190
134
 HER2
250
190
134
 EGFR
MW (kDa)
MW (kDa)
46
32
 β-Actin
46
32
 β-Actin

## Slide 2
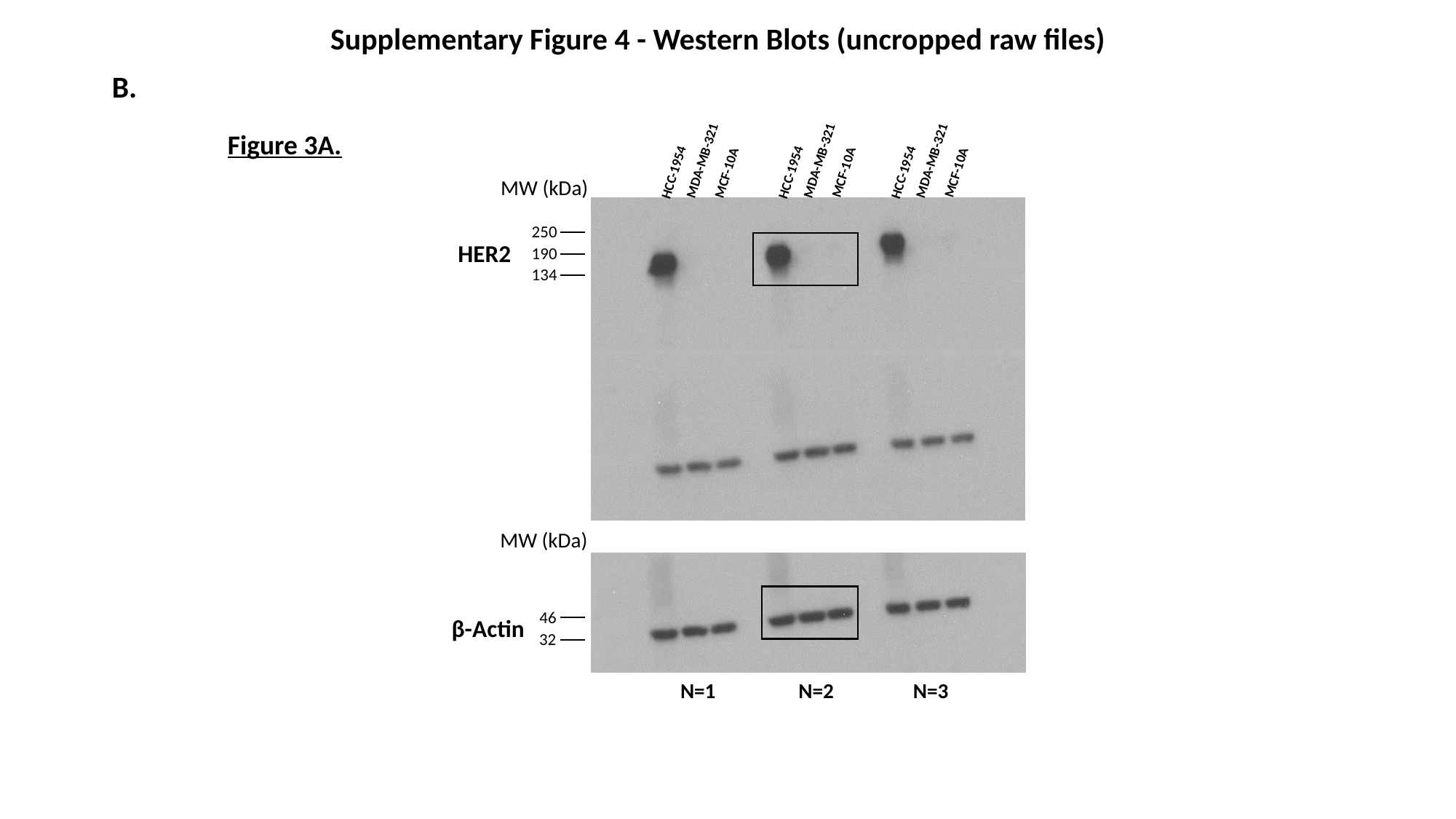

Supplementary Figure 4 - Western Blots (uncropped raw files)
B.
MDA-MB-321
MCF-10A
HCC-1954
MDA-MB-321
MCF-10A
HCC-1954
MDA-MB-321
MCF-10A
HCC-1954
MW (kDa)
250
190
134
 HER2
MW (kDa)
46
32
 β-Actin
N=1
N=2
N=3
Figure 3A.

## Slide 3
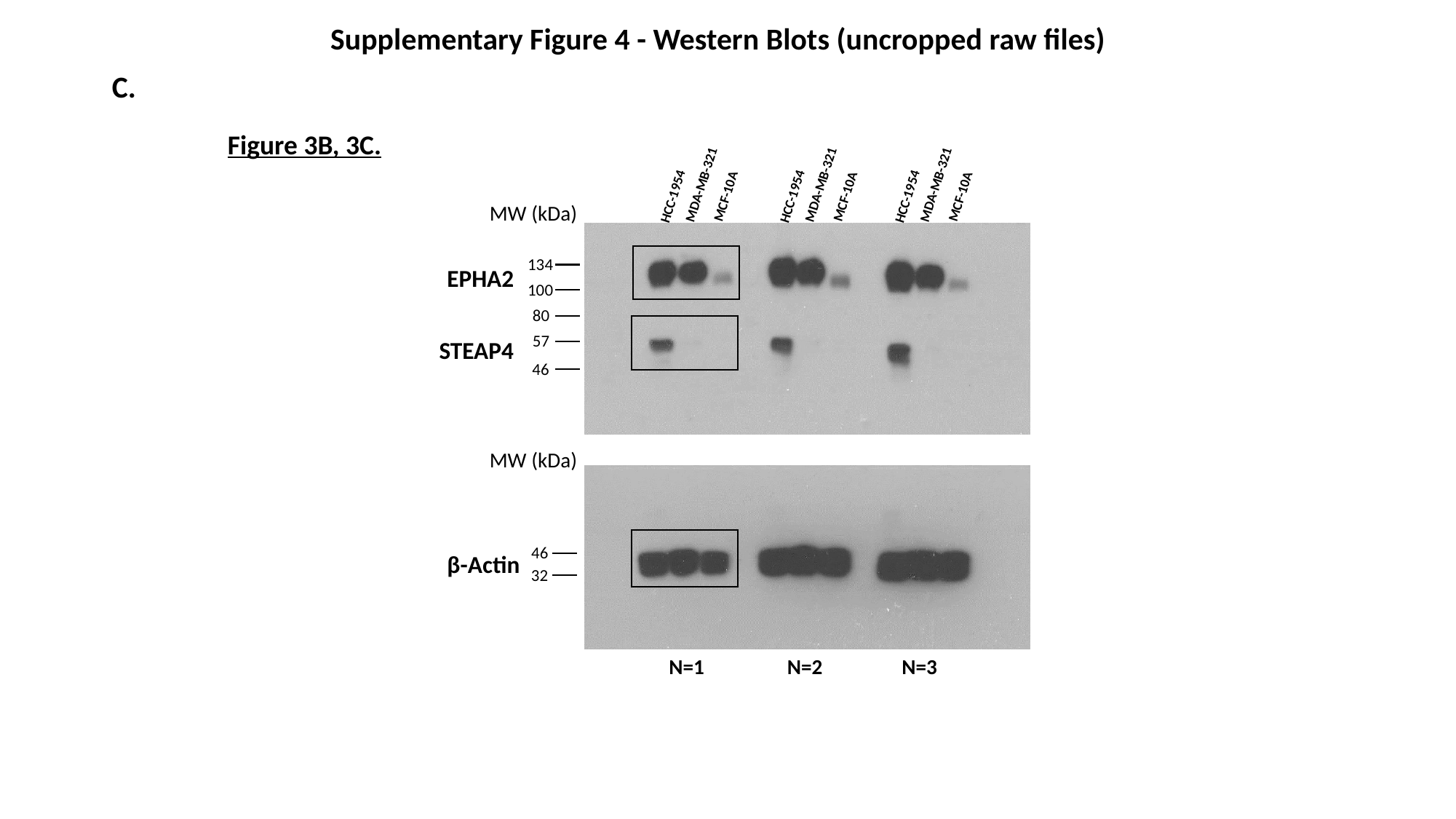

Supplementary Figure 4 - Western Blots (uncropped raw files)
C.
Figure 3B, 3C.
MDA-MB-321
MCF-10A
HCC-1954
MDA-MB-321
HCC-1954
MCF-10A
MDA-MB-321
HCC-1954
MCF-10A
MW (kDa)
134
100
80
57
46
 EPHA2
STEAP4
MW (kDa)
46
32
 β-Actin
N=1
N=2
N=3

## Slide 4
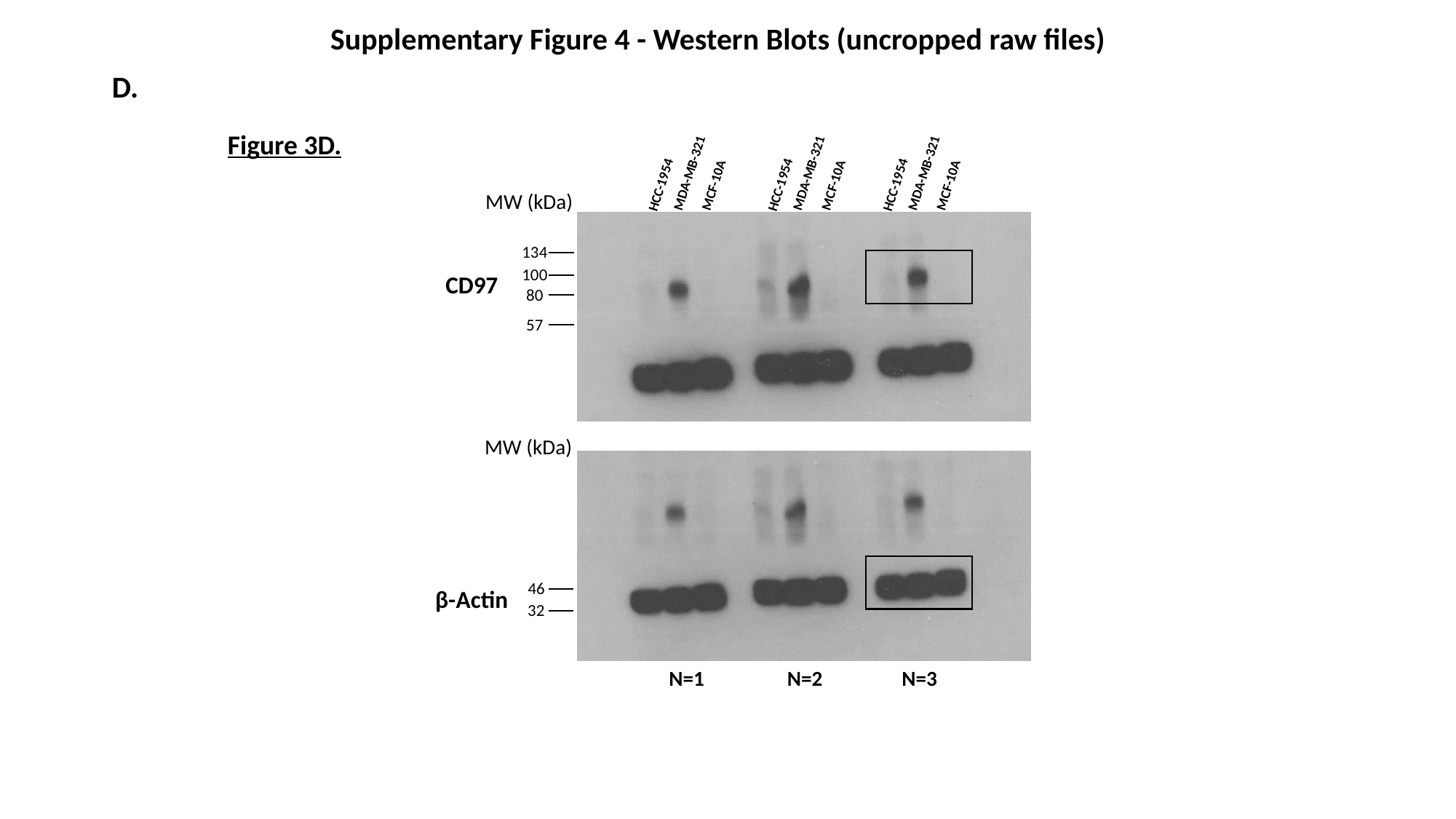

Supplementary Figure 4 - Western Blots (uncropped raw files)
D.
Figure 3D.
MDA-MB-321
HCC-1954
MCF-10A
MDA-MB-321
HCC-1954
MCF-10A
MDA-MB-321
MCF-10A
HCC-1954
MW (kDa)
134
100
80
57
CD97
MW (kDa)
46
32
β-Actin
N=1
N=2
N=3

## Slide 5
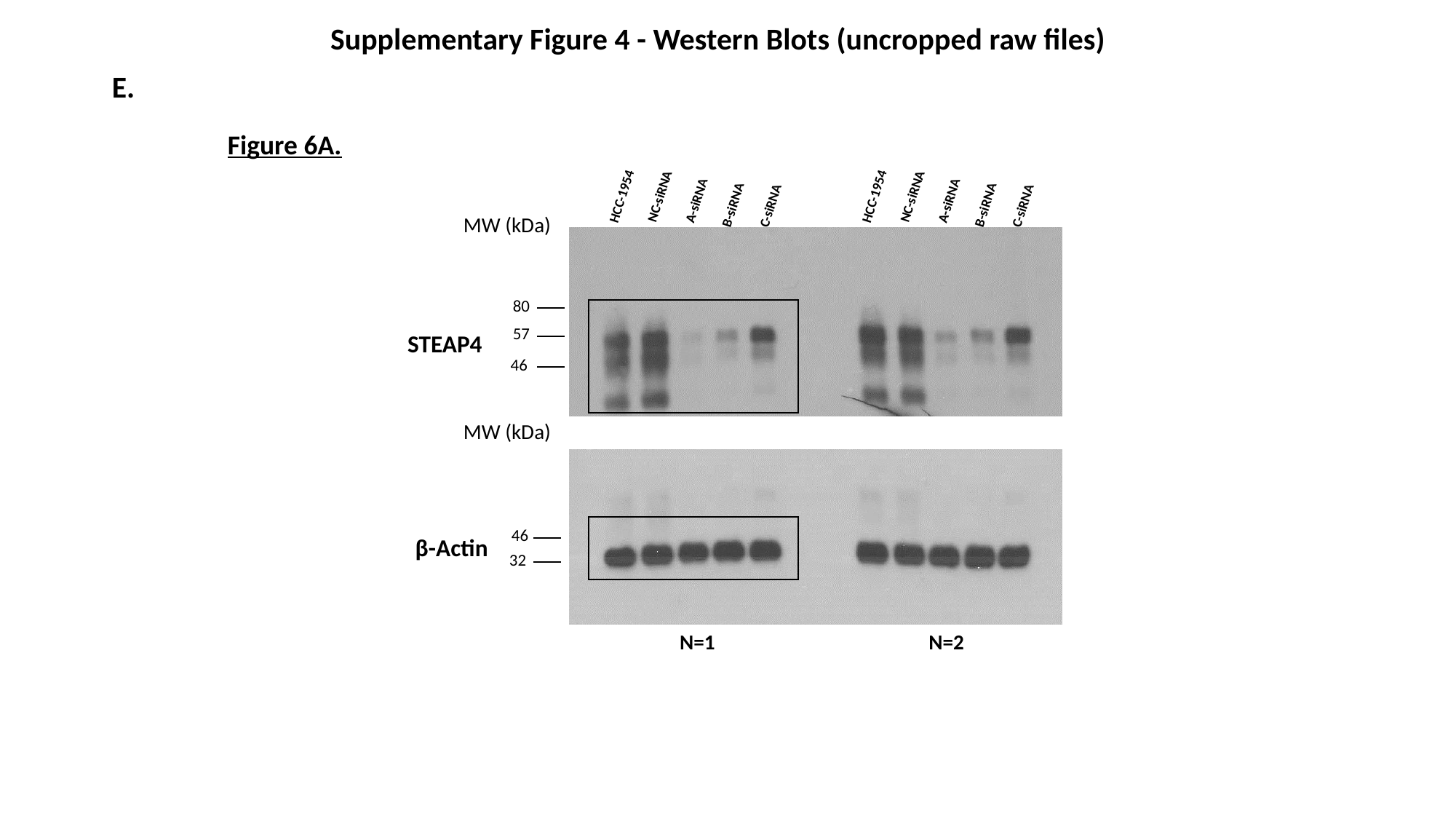

Supplementary Figure 4 - Western Blots (uncropped raw files)
E.
Figure 6A.
HCC-1954
NC-siRNA
A-siRNA
B-siRNA
C-siRNA
HCC-1954
NC-siRNA
A-siRNA
B-siRNA
C-siRNA
MW (kDa)
80
57
46
STEAP4
MW (kDa)
46
32
 β-Actin
N=1
N=2
